# Pesticide Residues in Egyptian Strawberries Inspected at the EU Border (2021–2024)

**DOI:** 10.3390/molecules30244780

**Published:** 2025-12-15

**Authors:** Kalina Maja Sikorska-Zimny, Artur Miszczak

**Affiliations:** 1 Laboratory of Postharvest Physiology of Horticultural Products, The National Institute of Horticultural Research, Pomologiczna 13b Street, 96-100 Skierniewice, Poland; 2 Food Safety Laboratory, The National Institute of Horticultural Research, Pomologiczna 13b Street, 96-100 Skierniewice, Poland; artur.miszczak@inhort.pl

**Keywords:** pesticide residues, strawberries, food safety, maximum residue level, EU regulation, African exports

## Abstract

The increasing reliance of the European Union on strawberry imports from North African countries, particularly Egypt, underscores the necessity of systematic monitoring of these commodities for pesticide residues prior to their placement on the EU market. This study evaluated pesticide residues in Egyptian strawberries inspected at the Polish border between 2021 and 2024. Detection rates rose sharply from 63% in 2022 to over 90% in 2023–2024, although a subset of samples each year contained no detectable residues (1 sample in 2021 and 2024; 2 in 2022), confirming that pesticide-free production is achievable. Fosetyl-aluminium was the most frequently identified compound, followed by bromide ion, azoxystrobin, and boscalid. Eleven exceedances of maximum residue levels (MRLs) were recorded, involving substances not approved in the EU due to carcinogenic, neurotoxic, or endocrine-disrupting properties. Multiple-residue presence was common, with up to eleven pesticides detected in a single sample. The findings highlight the need for broader surveillance, stricter enforcement, and support for sustainable pest management in exporting countries.

## 1. Introduction

The international fruit trade has expanded significantly in recent decades, driven by the rising consumer demand for fresh produce year-round. The European Union (EU) has become increasingly reliant on imports of soft fruits, including strawberries, particularly from African countries such as Egypt and Morocco, to meet the off-season demand [1]. While this trade provides substantial economic opportunities for exporters, it also introduces notable food safety challenges, particularly with regard to pesticide residues [2].

Strawberries are imported to the EU from North African countries, such as Morocco and Egypt [3]. In 2023, Egypt exported 25.43 million kg of fresh strawberries (3.62% increase from 2022) [4]. However, this growth has been accompanied by heightened scrutiny of pesticide residues, as the EU enforces some of the world’s most stringent food-safety regulations.

Pesticides are indispensable for crop protection in many agricultural systems; however, certain active substances are not approved in the EU because of toxicological risks. The EU’s Maximum Residue Levels (MRLs) for pesticides in food and feed, as established in Regulation (EC) No 396/2005, are designed to protect public health and the environment [5]. The EU’s border control system responds to non-compliant findings through reinforced checks, consignment rejection, and notification of Member States via the Rapid Alert System for Food and Feed (RASFF). Including this information highlights the regulatory consequences of repeated detections of not approved or unauthorised substances and strengthens the practical relevance of our findings for food-safety management. The Rapid Alert System for Food and Feed (RASFF) plays a critical role in monitoring and managing non-compliance, with pesticide-related notifications accounting for approximately one-third of all alerts in 2023 [2].

EU legislation explicitly classes several pesticides as “not approved”, including organophosphates such as chlorpyrifos, owing to evidence of neurodevelopmental toxicity, particularly in children [6]. Carbamates and specific neonicotinoids are also subject to strict restrictions because of their potential to cause neurotoxicity, endocrine disruption, and carcinogenicity [7,8]. Despite these prohibitions, many of these substances remain in use in non-EU countries, where regulatory frameworks and agricultural practices often differ significantly [9,10]. It should be noted that some pesticides classified as “not approved” in the European Union obtained this status not due to newly identified safety concerns, but because their authorization was not renewed. In many cases, this resulted from the fact that the manufacturers or stakeholders did not apply for renewal of approval.

This study contributes to the growing body of evidence on pesticide residues in imported produce, offering critical insights for policymakers, agricultural practitioners and public health authorities. By elucidating temporal trends and identifying compounds of concern, this study aims to inform targeted interventions that safeguard both consumer health and the integrity of the global food supply chain.

## 2. Results

**Figure 1:**
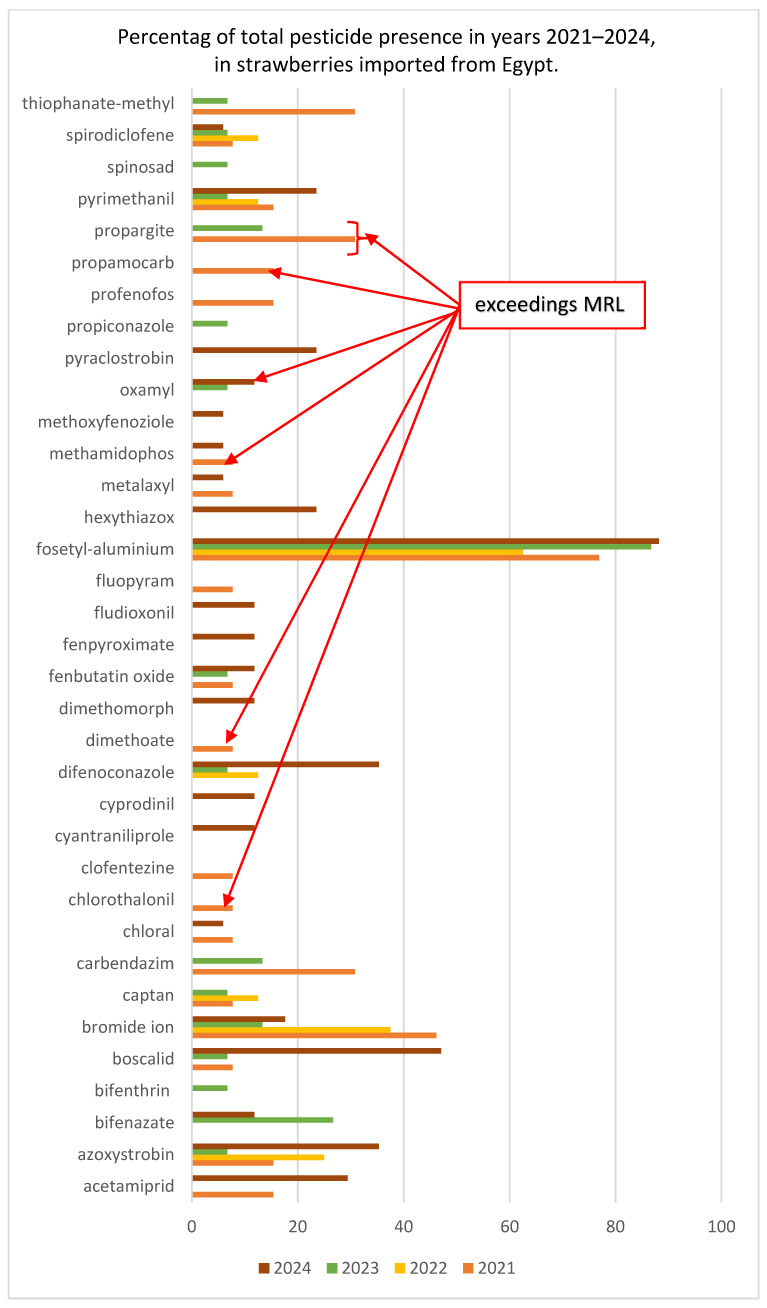
Pesticide presence in strawberries imported from Egypt (2021–2024). Clofentezine and oxamyl had an expiry date of 2023.

In this study, it was presented an analysis of pesticide residues in strawberries imported from Egypt to Europe over a four-year period. As shown in Figure 1 and Supplementary Table S1, the most frequently detected pesticide was fosetyl-aluminium (43 samples), followed by bromide ion (14), azoxystrobin (11), and boscalid (10), whereas all other substances (if) were detected in fewer than 10 samples. The compounds exceeding the maximum residue limits (MRLs) included chlorothalonil (2021), dimethoate (2021), methamidophos (2021), oxamyl (2024), propamocarb (2021), and propargite (2021 and 2023). Numerical exceedances of Maximum Residue Levels (MRLs) do not constitute legal non-compliances. MRLs are regulatory thresholds established by expert scientific bodies to ensure consumer protection and include conservative safety margins. Their role is to support risk-based official controls, and therefore exceedances should not be interpreted as equivalent to batches being legally non-compliant. The data also indicates a dynamic shift in pesticide usage patterns: while the detection of bromide ions declined systematically, the occurrence of boscalid, azoxystrobin, and acetamiprid surged in 2024, reflecting potential changes in pest-management strategies. Furthermore, 2022 was an outlier, characterised by the lowest detection frequency and the highest proportion of residue-free samples, in contrast to 2024, which exhibited the broadest spectrum of detected compounds and the emergence of previously undetected substances.

The graph presents a summary of the detections of specific pesticides from 2021 to 2024. Compounds exceeding the MRLs are marked in red (Figure 1).

It should also be emphasized that in 2021, 2022, and 2024, there were samples in which no pesticide residues were detected.

Fosetyl-aluminium was the most frequently and consistently detected pesticide across all study years. Notably, detections of boscalid, azoxystrobin, and acetamiprid markedly increased in 2024, suggesting a recent intensification in the use of fungicides and insecticides. Two distinct temporal patterns can be highlighted. In 2022—despite the smallest sample size—the data revealed the lowest overall detection frequency, reduced pesticide diversity, and the highest proportion of samples free from residues (*n* = 3). Importantly, no maximum residue level (MRL) exceedances were recorded during this year.

Conversely, 2024 was characterised by the broadest spectrum of detected compounds. Several previously rare substances (less frequently determined/detected in earlier samples e.g., acetamiprid, metalaxyl, and methamidophos) showed an increase in frequency, while additional compounds not detected in earlier years emerged, including cyprodinil, cyantraniliprole, dimethomorph, fenpyroximate, fludioxonil, hexythiazox, methoxyfenoziole and pyraclostrobin.

## 3. Discussion

As shown in the above analyses, the changes in pesticide use in individual years are quite significant. This may be influenced not only by climatic conditions, and thus the protection against fungal diseases or insects, but also by the availability of specific chemical substances that constitute pesticide. The modification of a name, manufacturer, or market presence will be another factor limiting or supporting the use of a given pesticide. Legal aspects arising from regulations concerning the use/application of preparations on the European market should not be overlooked. Exclusion from use within the EU results in restrictions on the application of a given compound by producers planning to sell to the EU, with a simultaneous possibility of shifting the market for the preparation already not approved in the EU to other regions.

The concern, as well as grounds for immediate market withdrawal, arises from samples in which analyses revealed exceedances of maximum residue levels (MRLs). Over four years, 11 exceedances were identified in nine samples. In the most recent two years, these cases involved oxamyl (2024) and propargite (2023). Another major concern is the detection of multiple residues. In the most extreme case (*n* = 1), as many as 11 different compounds were identified within a single sample, and ten compounds were detected in two additional samples. Although several pesticides in the analysed samples exceeded their respective MRLs, this exceedance should not be interpreted as an automatic indicator of adverse health effects. MRLs are not toxicological safety thresholds; rather, they represent the highest residue level expected when a pesticide is used according to the EU-authorised critical Good Agricultural Practice (GAP). Numerical exceedances of MRLs reported in this study do not imply legal non-compliance, as formal non-compliance can only be determined by the competent authorities, in which case the batch from which the sample originates cannot be placed on the market.

The data showed an increase in pesticide detection rates over the study period. Detection rates nearly doubled from 2022 (63%) to 2023 (93%), and in 2024, the high detection rate was maintained at 94%. Also, Malhat et al. (2025) in detection of pesticide residues in Egyptian strawberries pointed on the increasing level of pesticides usage from 65,55% in 2023 to 75.85% in 2024 [11]. However, Salim et al. (2019) detected no exceedance of MRL’s in strawberries from the year 2019 [12].

The most frequently detected pesticide was fosetyl-al (fungicide), consistently identified across all study years. It was detected in 10 of 13 samples in 2021, 5 of 8 samples in 2022, 13 of 15 samples in 2023, and 15 of 17 samples in 2024. Such persistent detection indicates widespread fungicide application. The second most frequently detected pesticide was bromide ion. It needs to be highlighted that bromide ion is not used as a plant protection product; it occurs naturally in the environment and may reflect background bromide levels in crops. Although methyl bromide (pesticide, fumigant) itself cannot be quantified by control authorities, it is naturally transformed into bromide ion, for which MRLs are established [13]. Therefore, the residues detected in strawberries most likely represent endogenous bromide rather than the result of any treatment. Third most detected substance was azoxystrobin (fungicide), which exhibited a notable increase in 2024, being present in 7 of 17 samples. The fourth was boscalid (fungicide), detected at significant levels in 2024 (8 of 17 samples), with only minimal detection in earlier years, suggesting recent adoption or increased use. Also, Malhat et al. (2025) noticed increase in pesticides detection wit most frequently during 4-year study detected azoxystrobin, boscalid, difenoconazole, fludioxonil and bifenazate [11].

Several pesticides exceeded maximum residue levels (MRLs), raising potential health concerns. In 2021, five pesticides were detected above their respective MRLs: chlorothalonil, dimethoate, methamidophos, propamocarb (one sample), and propargite (four samples). In 2023, propargite exceeded the MRL in one sample, while in 2024, oxamyl exceeded the MRL in two samples. The high detection frequency, combined with multiple MRL exceedances, poses serious food safety risks. Propargite, which exceeded the MRL in several samples, and has not been approved in the EU [14]. In the 2011 EFSA peer review, propargite raised several concerns due to data gaps in the toxicological assessment. The substance demonstrated carcinogenic potential in two rat strains, affecting different organs, and initial genotoxicity data were insufficient to exclude a genotoxic mode of action, leading to a critical area of concern [15]. Propargite was also classified for causing serious eye damage and toxicity by inhalation (R41; R23), potential skin sensitization (R43), serious health damage from prolonged oral exposure (R48/22), and possible risk to the unborn child (R63), with limited evidence of carcinogenic effects (R40) [16,17].

However, the 2018 EFSA opinion on setting MRLs for propargite in citrus fruits and tea [18] included a comprehensive reassessment of the toxicological data package, which addressed many of the previously identified data gaps. New valid genotoxicity studies demonstrated that propargite technical material is unlikely to be genotoxic in vivo, thereby excluding a genotoxic mode of action for the observed tumours. Although carcinogenic potential remains in rats, EFSA established toxicological reference values and determined that consumer exposure through dietary intake at the proposed MRL levels would not pose a health concern. The chronic exposure assessment used median residue (STMR) values from supervised trials, while acute exposure was evaluated using the highest residue (HR) level for oranges and STMR for tea, incorporating appropriate processing factors (peeling factor for oranges) and a variability factor of 7 for oranges to account for inhomogeneous distribution. The risk assessment concluded that adverse effects occur only at exposure levels significantly higher than those relevant for dietary intake at the established MRLs. Although it is a lipophilic compound, as indicated by its log Kₒw value, EFSA has not reported evidence of bioaccumulation or persistence in fatty tissues [18]. Importantly, EFSA has established consumer-safe MRLs for citrus fruits and tea, indicating that adverse effects occur only at exposure levels significantly higher than those relevant for dietary intake.

Organophosphate pesticides, including dimethoate and methamidophos, were also identified. These compounds predominantly act through inhibition of acetylcholinesterase, disrupting normal nervous system function [19]. Acute exposure may lead to nausea, vomiting, headaches, muscle fasciculations, and respiratory distress; severe intoxication can result in seizures, respiratory failure [20]. Chlorothalonil is classified as a probable human carcinogen and has been linked to kidney tumours [21]. It also causes respiratory irritation and may suppress immune function. Oxamyl is a carbamate insecticide and nematicide. Unlike organophosphates, carbamates are reversible acetylcholinesterase inhibitors, but they disrupt neurotransmission in a similar way [22]. In humans, acute exposure can cause cholinergic symptoms, including headache, dizziness, confusion, muscle fasciculations, seizures, and respiratory depression. Cardiovascular manifestations include bradycardia, hypotension, and arrhythmias. Gastrointestinal effects include nausea, vomiting, diarrhoea, and abdominal cramps, whereas respiratory complications may involve bronchospasm, hypersalivation, and pulmonary oedema [23]. Epidemiological studies suggest that prenatal carbamate exposure may contribute to neurodevelopmental delays in children, including impaired cognition and behavioural abnormalities [24]. Chronic exposure is associated with immunosuppression, increased infection susceptibility, and potentially reduced vaccine efficacy. Propamocarb, which exceeded the MRL in one sample in 2021, can cause gastrointestinal irritation, transient neurological symptoms such as weakness and impaired coordination, and respiratory irritation upon inhalation exposure [25,26]. Experimental studies have indicated potential hepatotoxicity, nephrotoxicity, thyroid dysfunction, and possible disruption of reproductive hormone regulation [27].

Exceeding maximum residue levels (MRLs) is an important issue; however, an equally important concern lies in the presence of pesticides with well-documented adverse effects on human health (often already not approved in the EU), as well as in the overall mixture of pesticides found in a single sample. Multiple pesticide exposure—the so-called cocktail effect—represents a significant health threat because combined toxicological outcomes may exceed the sum of individual risks. In particular, synergistic interactions can occur where one pesticide enhances the toxicity of another (e.g., organophosphates combined with carbamates). Despite this, research on the health effects of pesticide mixtures remains limited [28,29].

The magnitude and type of threat vary across populations. In children, higher absorption rates, stemming from an increased surface-area-to-body-weight ratio, result in greater pesticide uptake [30]. Moreover, their developing nervous, immune, and reproductive systems are particularly susceptible to disruption. Emerging evidence suggests that pesticide exposure may also contribute to neurodevelopmental and behavioural disorders, such as attention-deficit/hyperactivity disorder (ADHD), learning disabilities, and autism spectrum disorders [31]. For pregnant women, exposure to pesticide mixtures is especially dangerous during the first trimester, when critical organogenesis occurs [32]. Most pesticides can cross the placental barrier, potentially impairing foetal development and leading to adverse outcomes such as low birth weight, preterm delivery, and congenital malformations [33]. In elderly populations, the toxic effects of pesticide mixtures are amplified by age-related decline in liver and kidney function, as well as by comorbidities and polypharmacy, which are prevalent in older age groups (e.g., diabetes, cardiovascular diseases) [34].

Nevertheless, it must be recognized that the quantity of food products consumed containing residual pesticides is a decisive determinant of risk, since only sustained intake at high levels is likely to constitute a significant hazard to human health.

## 4. Materials and Methods

### 4.1. Samples

In subsequent years, strawberry samples delivered to the EU border from Egypt were also analysed. In 2021, 13 samples were collected, followed by 8 in 2022, 15 in 2023, and 17 in 2024. Imported fruits were sampled under the supervision of the Sanitary Inspection, a governmental authority overseeing food safety. Data collection took place at border checkpoints and in warehouses distributed across the country. Samples of imported food were collected by the Sanitary Inspection, the national authority responsible for food supervision in Poland. The materials were obtained at border checkpoints. All plant products met the visual quality and formal criteria for food transport and marketing; no mechanical damage, pest infestation symptoms, or other surface defects were observed.

Within 48 h of sampling, all materials were delivered to the laboratory, where their integrity was verified and confirmed as appropriate for pesticide residue determination. Upon receipt, the samples were immediately cooled to −18 °C and homogenised using dry ice. The procedures for sample handling and analytical testing followed the standard practices and regulatory guidelines established for official laboratories in the European Union [35].

### 4.2. Methods

The analytes were classified into major functional groups according to their chemical structure and mode of action: insecticides (organochlorines, organophosphates, pyrethroids, carbamates, and others), fungicides (including dithiocarbamates and carbamates), herbicides, growth regulators, and other categories. A detailed list of the compounds and corresponding analytical methods is provided in Supplementary Tables S2–S5.

All procedures were accredited. The following instruments were employed for the pesticide residue analysis:

Gas chromatography with mass spectrometry (GC-MS/MS): Agilent 6850 Series + 5973 MSD (dithiocarbamates) (Santa Clara, CA, USA, purchased in Poland); Agilent 6890N + 5975B inert XL MSD (ethylene oxide) (Wilmington, DE, USA, purchased in Poland); Agilent 7890A + 7000 GC/MS Triple Quad (multimethod) (Shanghai, China, purchased in Poland); and Agilent 7890B + 7000D GC/MS Triple Quad (multimethod) (Shanghai, China, purchased in Poland).

Liquid chromatography with mass spectrometry (LC-MS/MS): Agilent HPLC 1200 Series + 6410 Triple Quad LC/MS (multimethod) (Waldbronn, Germany, purchased in Poland); Agilent HPLC 1260 Infinity + 6460 Triple Quad LC/MS (multimethod) (Waldbronn, Germany, Singapore, purchased in Poland); Agilent HPLC 1260 Infinity II + 6470A Triple Quad LC/MS (QuPPe) (Waldbronn, Germany, purchased in Poland); and Agilent HPLC 1290 Infinity II + 6470B Triple Quad LC/TQ (QuPPe) (Waldbronn, Germany, purchased in Poland).

#### 4.2.1. PN-EN 15662:2018—GC-MS/MS and LC-MS/MS

This method is based on the QuEChERS approach, involving acetonitrile extraction, dispersive solid-phase clean-up (d-SPE), and subsequent GC-MS/MS and LC-MS/MS analysis. This method enables the determination of up to 499 pesticide residues. Sample preparation involved weighing 10 g of material into a 50 mL polypropylene tube, adding 10 mL of acetonitrile, vortexing for ~3 min, and supplementing with a buffer–salt mixture (4 g MgSO_4_, 1 g NaCl, 1 g Na-citrate dihydrate, 0.5 g Na-hydrogen citrate). After 3 min of shaking and centrifugation (7200 rpm, 5 min, RT), 1 mL of the extract was purified with MgSO_4_ and primary-secondary amine (PSA). The final extracts were analysed using GC-MS/MS and LC-MS/MS.

GC-MS/MS: Extracts were analysed using an Agilent 7890A GC (Agilent, Santa Clara, CA, USA) coupled with a 7000 Triple Quadrupole MS equipped with a DB-5MS column. Detection and quantification were performed in the MRM mode. LC-MS/MS: Extracts were analysed using an Agilent 1200 LC (Agilent, Santa Clara, CA, USA) with a 6410 Triple Quadrupole MS. Separation was achieved on an Eclipse Plus C18 column using gradient elution (Agilent Scientific Instruments, Santa Clara, CA, USA) with water (5 mM ammonium formate + 0.01% formic acid) and acetonitrile: water (95:5, *v*/*v*, with additives). Analyses were performed in positive ESI mode with Dynamic Multiple Reaction Monitoring (dMRM) acquisition. LOQs were set at 0.01 or 0.005 mg·kg^−1^, depending on the analyte (Supplementary Tables S2–S5).

##### Sub-Method 1—PSA-Free QuEChERS Variant

Sample extraction was identical to PN-EN 15662:2018, but without PSA purification. LC-MS/MS analysis was performed. The method covers the compounds listed in Supplementary Tables S2–S5 with an LOQ of 0.005 mg·kg^−1^.

##### Sub-Method 2—Acidic Herbicides (Modified PN-EN 15662:2018)

For acidic herbicides, the QuEChERS extraction method was modified by including alkaline hydrolysis. After extraction with acetonitrile, water, and NaOH, the mixture was neutralised with H_2_SO_4_ and formic acid, followed by salt addition and centrifugation. The extracts were then analysed by LC-MS/MS (Agilent 1260 + 6460 Triple Quad) on an Eclipse Plus C18 column. Detection was performed in both positive and negative ESI modes using the MRM. It was optimized explicitly for determining 33 acidic herbicides with an LOQ of 0.01 mg·kg^−1^

### 4.3. PN-EN 12396-2:2002—Dithiocarbamates

Dithiocarbamate residues were determined as carbon disulfide (CS_2_) using headspace GC with either FPD or MS detection. The method follows PN-EN 12396-2:2002, with a modification involving CS_2_ absorption in isooctane. Analyses were carried out on an Agilent 6850 GC with a 5973N MS detector. The LOQ was 0.01 mg·kg^−1^ (Supplementary Table S5).

### 4.4. QuPPe Method—Highly Polar Pesticides

Highly polar compounds (e.g., glyphosate and AMPA) were analysed using the QuPPe protocol. Extraction involved acidified methanol, with EDTA added for cereals, pulses, nuts, and oilseeds. The extracts were centrifuged, filtered, and directly analysed using LC-MS/MS (Agilent 1200 + 6460 Triple Quad).

Quantification relied on isotope-labelled internal standards added at the beginning of the procedure. LOQs were set at 0.01 mg·kg^−1^ (Supplementary Tables S2–S5). Results were reported according to SANTE/10704/2021 requirements.

## 5. Conclusions

The monitoring of Egyptian strawberry imports to the EU revealed pesticide residues, including repeated exceedances of maximum residue levels (MRLs) and the presence of multiple residues within single samples. Particularly concerning are the detections of propargite, oxamyl, and organophosphates such as dimethoate and methamidophos, substances already not approved in the EU due to their neurotoxic, carcinogenic, or endocrine-disrupting properties. The sharp rise in detection rates, from 63% in 2022 to over 90% in 2023–2024, indicates intensified pesticide use and highlights persistent weaknesses in compliance with EU food safety standards. The frequent occurrence of fungicides such as fosetyl-al, azoxystrobin, and boscalid points to widespread chemical reliance in Egyptian strawberry production.

Exceedances of MRLs, combined with the presence of multiple residues, raise concerns about potential cumulative effects on consumer health. In 2023, EFSA’s Pesticides Peer Review Panel outlined three methods to assess the combined toxicity of multiple active substances: addition of responses, addition of doses, and interaction. The Panel concluded that response addition is not relevant for pesticide residue risk assessment, and that synergistic interactions are highly unlikely at the low exposure levels typically encountered by consumers [36]. Nevertheless, vulnerable groups such as children, pregnant women, and the elderly warrant particular attention, as chronic exposures—even at low levels—may contribute to developmental, reproductive, or immunological effects. While current regulatory frameworks in the EU have begun implementing cumulative risk assessment for pesticide groups sharing a common mechanism of toxicity, further refinement is needed to comprehensively address the toxicity of complex mixtures across different modes of action.

This study has several limitations that should be considered when interpreting the findings. First, the dataset reflects only strawberries sampled at the EU border in Poland, which does not allow determination of the final destination markets within the European Union. Consequently, the results cannot be directly extrapolated to consumer exposure across specific Member States. Second, the number of analysed samples was relatively small, and sampling was dependent on official control procedures than a structured, statistically designed monitoring programme; therefore, the results may not fully represent the entire spectrum of pesticide use in Egyptian strawberry production. Third, only residue levels at the point of import were assessed, without information on pre- or post-harvest practices, environmental conditions, or storage and transport factors that may influence pesticide degradation. Finally, although multiple residues were detected, the study did not include a cumulative risk assessment or toxicological modelling of mixture effects, limiting the ability to draw conclusions regarding combined exposures. These constraints highlight the need for broader, coordinated monitoring and more comprehensive risk-assessment approaches to fully evaluate the safety implications of imported produce.

From a public health and regulatory perspective, these findings underscore the necessity of sustained surveillance, broader pesticide screening panels, and stricter enforcement measures, including temporary restrictions on high-risk consignments. Capacity-building for Egyptian producers, with emphasis on integrated pest management (IPM), organic alternatives, and sustainable cultivation practices, is essential to reduce reliance on hazardous substances. Finally, improved labelling and consumer awareness are needed to mitigate exposure risks and strengthen food safety in the EU market.

## Data Availability

The data presented in this study are available from Artur Miszczak upon request due to the large amount of measurement data obtained from analytical instruments.

## Supplementary Materials

The following supporting information can be downloaded at: https://www.mdpi.com/article/10.3390/molecules30244780/s1, Table S1: Detection of certain pesticides in each strawberry samples imported from Egypt; Table S2: Analytical methods were applied to specific product groups; Table S3: Classification of analytical methods by technique, target pesticide, and LOQ (according to SANTE: groups 1,2,3, e.g., fruits, vegetables, mushrooms, juices, etc.); Table S4: The list of the compounds and corresponding analytical method; Table S5: The list of the analytical methods details.
