# Supplementary material for: Pesticide Residues in Egyptian Strawberries Inspected at the EU Border (2021–2024)
Source: Molecules. 2025 Dec 15;30(24):4780. doi: 10.3390/molecules30244780 (PMC12736362; doi:10.3390/molecules30244780)
Supplement: Supplementary file 1 [file molecules-30-04780-s001.zip › molecules-3994926-supplementary.pdf]

SUPPLEMENTARY TABLES

Table S1. Detection of certain pesticides in each strawberry sample imported from Egypt.

year	samples number	acetamiprid	azoxystrobin	bifenazate	bifenthrin	boscalid	bromide ion	captan	carbendazim	chloral	chlorothalonil	clofentezine	cyantraniliprole	cyprodinil	difenoconazole	dimethoate	dimethomorph	fenbutatin oxide	fenpyroximate	fludioxonil	fluopyram	fosetyl-aluminium	hexythiazox	metalaxyl	methamidophos	methoxyfenozide	oxamyl	profenofos	propamocarb	propiconazole	propargite	pyraclostrobin	pyrimethanil	spinosad	spirodiclofene	thiophanate-methyl	total sum per sample
**2021**	1.					0,029	0,27															1,01															**3**
	2.	0,007																				0,88									***0,07***						**3**
	3.															***0,023***						0,32			***0,029***												**3**
	4.																																				**0**
	5.								0,006													0,36						0,006	0,01							0,007	**5**
	6.								0,054		***0,057***										0,006	3,5						0,005			***0,058***		0,034			0,075	**8**
	7.						1,6			0,01												2,6													0,011		**4**
	8.	0,008							0,009													5,7									***0,044***					0,019	**5**
	9.						0,92																														**1**
	10.		0,024				1,2	0,39	0,012			0,012						0,006				1,1		0,01					***0,072***				0,015			0,007	**11**
	11.						0,71															0,98									***0,039***						**3**
	12.						0,46															3,6															**2**
	13.		0,024																																		**1**
**2022**	1.																																				**0**
	2.																					0,24															**1**
	3.		0,028				0,8	0,061							0,038							3,4											0,16				**6**
	4.																					7,34															**1**
	5.		0,006				0,62															1,07													0,012		**4**
	6.																																				**0**
	7.																					0,64															**1**
	8.						0,35																														**1**
**2023**	1.		0,007					0,038							0,008							1,1															**4**
	2.						0,3		0,01													0,83															**3**
	3.																					1,4															**1**
	4.			0,014																		1,7															**2**
	5.																	0,007				0,74															**2**
	6.																					1,4					0,008								0,046		**3**
	7.																					2,6															**1**

	8.			0,013																		0,62															2
	9.																					0,16															1
	10.								0,012													0,16								0,01	0,015					0,005	5
	11.			0,018																		0,46									***0,029***						3
	12.						0,35																														1
	13.																					0,62															1
	14.			0,007																		0,6											0,006				3
	15.				0,013	0,01																												0,026			2
	1.																					1,1													0,15		2
	2.	0,016					0,64								0,024							0,96	0,005				***0,01***										6
	3.												0,036									0,75	0,011														3
	4.																					0,54															1
	5.	0,038				0,013								0,013					0,007	0,007		0,25															6
	6.																					1,1															1
	7.		0,008			0,01	0,8								0,015			0,01				2,7											0,062		0,006		8
	8.					0,006												0,015				2,8											0,079				4
2024	9.	0,016	0,006			0,032	0,56								0,017		0,018					4,3		0,006		0,008						0,013					10
	10.																					1,6															1
	11.																					0,63															1
	12.																																				0
	13.		0,005							0,014			0,026		0,01							2,8											0,024				6
	14.	0,037	0,036	0,012		0,006									0,11		0,014					1,1	0,007									0,008	0,024				10
	15.		0,023	0,012		0,046								0,008	0,011					0,007		6,3					***0,021***					0,019					9
	16.	0,053				0,082													0,007			0,9	0,014									0,018					6
	17.		0,034			0,01																															2

Values: bold and italicized indicate samples in which the specified pesticide exceeded the MRL

Table S2. Analytical methods were applied to specific product groups.

Food, agricultural products, plant material		
Object of study	Analitycal method	Determined substances (LoQ given in the table 3)
Products with high water content, high acid and water content, and high sugar and low water content (SANTE: groups 1, 2, 3)<br><br>e.g.:<br>- fresh fruits<br>- fresh vegetables<br>- fresh herbs<br>- fresh mushrooms<br>- juices and concentrates<br>- honey<br>- dried fruits<br>- jams	PN-EN 15662:2018	2-phenylphenol, acetochlor, acrinathrin,alachlor,aldrin,allethrin, ametryn,aminocarb,anthraquinone,atrazine,azaconazole, azinphos ethyl, azinphos-methyl, azoxystrobin, beflubutamid, benalaxyl, benfluralin, benfuracarb, bifenazate, bifenazate diazene, bifenox, bifenthrin, biphenyl, bitertanol, boscalid, bromfenvinphos, bromocyclen, bromophos, bromophos-ethyl, bromopropylate, bupirimate, buprofezin, butachlor, butafenacil, butylate, captafol,captan,carbaryl,carboxin,chinomethionat, chlorbenside, chlorbufam,chlordane.-cis,chlordane.-oxy,chlordane-trans, chlorfenapyr, chlofens on, chlorfenvinphos, chlormephos, chlorobenzilate, chloropropylate, chlorothalonil, chlorpropham, chlorpyrifos, chlorpyrifos-methyl, chlorthal-dimethyl, chlorthion, chlorthiophos, clodinafop propargyl, clomazone, coumaphos, crimidine, cyanazine, cyanophenphos, cyanophos, cycloate, cyfluthrin, cypermethrin, cyprazine, cyproconazole, cyprodinil, ddd-o,p', ddd-p,p’, dde-o,p’, dde-p,p’, ddm, ddt-o,p', ddt-p,p', deltamethrin, demeton-s, desmetryn, dialifos, diazinon, dichlobenil, dichlofenthion, dichlofluanid, dichloroaniline, 3,5-, dichlorobenzamide, 2,6-, dichlorobenzophenone-p,p, dichlorvos, diclobutrazol, dicloran, dicofol, dieldrin, diethofencarb, difenoconazole, dimethachlor, dimethoate, dimethomorph, dimoxystrobin, diniconazole, dinitramine, dinobuton, dinoseb, dioxabenzofos, dioxacarb, dioxathion, diphenylamine, disulfoton, disulfoton sulfon, disulfoton sulfoxide, ditalimfos, dmst, dodemorph, edifenphos, endosulfan alpha, endosulfan beta, endosulfan sulphate, endrin, endrin keton, epn, epoxiconazole, esfenvalerate, etaconazole, ethalfluralin, ethion, ethofumesate, ethofumesate, -2-keto, ethoprophos, ethoxyquin, etofenprox, etrimfos, fenamiphos, fenarimol, fenazaquin, fenbuconazole, fenchlorphos, fenhexamid, fenitrothion, fenoxycarb, fenpropathrin, fenpropidin, fenpropimorph, fenpyrazamine, fenthion, fenvalerate, fipronil, fipronil desulfinyl, fipronil sulfon, fluchloralin, flucythrinate, fludioxonil, fluensulfone, flumetralin, flumioxazin, fluorodifen, fluotrimazole, fluquinconazole, flurprimidol, flurtamone, flusilazole, flutianil, flutriafol, folpet, fonofos, formothion, furalaxyl, furathiocarb, gamma-cyhalothrin, halfenprox, heptachlor, heptachlor cis-epoxid, heptachlor trans-epoxid, heptenophos, hexachlorobenzene, hexachlorocyclohexane (hch), alpha, hexachlorocyclohexane (hch), beta, hexaconazole, imazalil, iodofenphos, iprobenfos, iprodione, isocarbophos, isofenphos, isofenphos-methyl, isofetamid, kresoxim-methyl, lambda-cyhalothrin, lindane, malaoxon, malathion, mandestrobin, mecarbam, mepanipyrim, mepronil, metalaxyl, metazachlor, metconazole, methacrifos, methidathion, methoxychlor, metolachlor, metribuzin, mevinphos, molinate, myclobutanil, nitralin, nitrapyrin, nitrofen, nitrothal isopropyl, nuarimol, oxadiazon, oxadixyl, oxyfluorfen, paclobutrazol, parathion, parathion-methyl, penconazole, pencycuron,pendimethalin, penflufen, pentachloroaniline, permethrin, perthane (ethylan), pethoxamid, phenthoate, phorate, phorate sulfone, phorate sulfoxide, phosalone, phosmet, phosphamidon, phthalimide, picolinafen, picoxystrobin, piperonyl butoxide, piperophos, pirimicarb, pirimicarb desmethyl, pirimiphos-ethyl, pirimiphos-methyl, procymidone, profenofos, profluralin, prometon, prometryn, propachlor, propargite, propazine, propetamphos, propham, propiconazole, propyzamide, prothioconazole destio, prothiofos, pyraclostrobin, pyrazophos, pyrethrins, pyridaben, pyridalyl, pyridaphenthion, pyrifenox, pyrimethanil, pyriofenone, pyriproxyfen, pyroquilon, quinalphos, quinoxyfen, quintozene, resmethrin, silafluofen, simazine, spiromesifen, sulfotep, tau-fluvalinate, tebuconazole, tebufenpyrad, tecnazene, tefluthrin, terbacil, terbufos, terbutryn, tetrachlorvinphos, tetraconazole, tetradifon, tetrahydrophthalimide, tetramethrin, tetrasul, thiobencarb, tolclofos-methyl, tolylfluanid, triadimefon, triadimenol, tri-allate, triazophos, trifloxystrobin, triflumizole, trifluralin, vinclozolin
		abamectin, acephate, acetamiprid, aclonifen, aldicarb, aldicarb sulfone, aldicarb sulfoxide, ametoctradin, amidosulfuron, amisulbrom, azadirachtin, aziprotryne, azoxystrobin, bac c10, bac c12, bac c14, bac c8, beflubutamid, bendiocarb, benthiavalicarb isopropyl, benzovindiflupyr, bixafen, boscalid, bromacil, bromuconazole, cadusafos, carbaryl, carbendazim, carbetamide, carbofuran, carbofuran 3-hydroxy, carbofuran 3-keto, carfentr azone-ethyl, chlorantraniliprole, chloridazon, chlorotoluron, chlorpyrifos, chlorsulfuron, chromafenozide, clodinafop propargyl,clofentezine, clothi anidin, cyantraniliprole, cyazofamid, cycloxydim, cyflufenamid, cyflumetofen, cymiazol, cymoxanil, cyproconazole, ddac c8, deet, demeton-s-met hyl, demeton-s-methyl sulphone, demeton-s-methyl sulphoxide, desmedipham, dicrotophos, diethofencarb, diflubenzuron, diflufenican, dimethena mid, dimethoate, dinotefuran, disulfoton, disulfoton sulfon, disulfoton sulfoxide, diuron, dmf, dmpf, emamectin b1a, emamectin b1b, ethametsulfu ron-ethyl, ethiofencarb, ethirimol, etoxazole, famoxadone, fenamidone, fenamiphos, fenamiphos sulfoxide, fenamiphos sulphone, fenbuconazole, f

		enfuram, fenhexamid, fenobucarb, fenoxaprop-p-ethyl, fenpropidin, fenpropimorph,fenpyroximate, fensulfothion, fensulfothion oxon, fensulfothion oxon sulphone, fensulfothion sulphone, fenthion, fenthion oxon, fenthion oxon sulphone, fenthion sulfoxide, fenthion sulphone, flazasulfuron, flonicamid, florasulam, flufenacet, flufenoxuron, fluopicolide,fluopyram, fluoxastrobin, flupyradifurone, flurochloridone, flutianil, flutolanil, flutriafol, fluxapyroxad, foramsulfuron, formetanate, fosthiazate, fuberidazole, halofenozide, hexaflumuron,hexythiazox, imazalil, imazapic, imidacloprid, indoxacarb, iodosulfuron methyl, ipconazole, iprovalicarb, isofetamid, isoprocarb, isoprothiolane, isoproturon, isopyrazam, isoxaben, isoxaflutole, isoxathion, lenacil, linuron, lufenuron, malaoxon, malathion, mandipropamid, metaflumizone,metalaxyl, metamitron, metazachlor,methamidophos, methiocarb, methiocarb sulphone, methiocarb sulphoxide, methomyl, methoprotryne, methoxyfenozide, metobromuron, metolachlor-s, metosulam, metoxuron, metrafenone, metsulfuron-methyl, monocrotophos, monuron, napropamide, nicosulfuron, nicotine, nitenpyram, novaluron, omethoate, oxadixyl,oxamyl, oxycarboxin, paraoxon-methyl, parathion, parathion-methyl, pencycuron, pendimethalin, penflufen, penthiopyrad, pethoxamid, phenmedipham, phenthoate, phosmet, phosmet oxon, phoxim, pinoxaden, piperonyl butoxide, prochloraz, prochloraz bts 44595, prochloraz bts 44596, propamocarb, propaquizafop, propoxur, propoxycarbazone, proquinazid, prosulfocarb, prosulfuron, pymetrozine, pyridaben, pyridafol, pyrifenox, pyriproxyfen, pyroquilon, pyroxsulam, quinclorac, quinoclamine, quizalofopethyl, rimsulfuron, rotenone, saflufenacil, silthiofam, spinetoram c42, spinetoram c43, spinosyn a, spinosyn d, spirodiclofen, spirotetramat, spirotetramat enol, spirotetramat enol-glucoside, spirotetramat ketohydroxy, spirotetramat monohydroxy, spiroxamine, sulfometuron methyl, sulfosulfuron, sulfoxaflor, tebuconazole, tebufenozide, tebufenpyrad, teflubenzuron, tepraloxydim, terbufos, terbufos oxon, terbufos sulphone, terbufos sulphoxide, terbuthylazine, thiabendazole, thiacloprid, thiamethoxam, thiencarbazone-methyl, thifensulfuron-methyl, thiodicarb, thiometon, thiophanate-methyl, tolfenpyrad, topramezone, tralkoxydim, trichlorfon, tricyclazole, tridemorph, triflumizole, triflumuron, triflusulfuron methyl, triticonazole, tritosulfuron, zoxamide
		2,4,5-t,2,4-2,4-db, acibenzolar acid, acifluorfen, aminopyralid, bentazone, bromacil, bromoxynil, clopyralid, dicamba, dichlorprop, diclofop, diflufenzopyr, fenoprop, fenoxaprop, fluazifop, fluroxypyr, haloxyfop, imazamox, imazapyr, imazethapyr, ioxynil, mcpa, mcpb, mecoprop, picloram, quinmerac, quizalofop, triasulfuron, tribenuron methyl, triclopyr, trinexapac
		cyhexatin, dithianon, dodine, fenbutatin oxide, fentin, flonicamid, TFNA TFNA-AM, TFNG
	PN-EN 12396-2:2002	dithiocarbamates expressed as CS_2
	PB-04 ed.1 of 10.01.2022	ethylene oxide (2-chloroethanol expressed as ethylene oxide)
	EURL-SRM QuPPe - PO	glyphosate, trimethylsulfonyl cation, glufosinate together with matabolites (MPP, N-acetyl-glufosinate), ethephon
		maleic hydrazide
		chlorates, perchlorates, phosetyl, phosphonic acid, bromide ion
		chlormequat chloride, mepiquat chloride, cyromazine, matrine, oxymatrine, melamine
		AMPA (not acreditated)
		ETU, PTU (not acreditated)

Table S3. Classification of analytical methods by technique, target pesticide, and LOQ (according to SANTE: groups 1,2,3 e.g. fruits, vegetables, mushrooms, juices, etc.).

Methods	Substance	LoQ [mg/kg]
GC-MS/MS	oxy-chlordane, trans-chlordane, cyprazine, 2,6-dichlorobenzamide, dinitramine, dinobuton, dinoseb, disulfoton sulfon, disulfoton sulfoxide, endrin keton, 2-keto ethofumesate, fenpyrazamine, fluensulfone,flumioxazin, flurprimidol, flurtamone, flutianil, isofetamid, mandestrobin, molinate, oxadiazon, penflufen, pentachloroaniline, phorate sulfone, pirimiphos-ethyl, pyridalyl, pyridaphenthion, silafluofen, simazine, thiobencarb	0.01

	2-phenylphenol, acetochlor, acrinathrin, alachlor, allethrin, ametryn, aminocarb, anthraquinone, atrazine, azaconazole, fenthion, fenvalerate, azinphos ethyl, azinphos-methyl, azoxystrobin, beflubutamid, benalaxyl, benfluralin, benfuracarb, bifenazate, bifenazate diazene, bifenox, bifenthrin, biphenyl, bitertanol, boscalid, bromfenvinphos, bromocyclen, bromophos, bromophos-ethyl, ethoprophos, ethoxyquin, etofenprox, etrimfos, fenamiphos, fenarimol, fenazaquin, fenbuconazole, fenchlorphos, fenhexamid, fenitrothion, fenoxycarb, fenpropathrin, fenpropidin, fenpropimorph, bromopropylate, bupirimate, buprofezin, butachlor, butafenacil, butylate, captafol, captan, carbaryl, carboxin, chinomethionat, chlorbenside, chlorbufam, chlordane, cis- chlorfenapyr, chlorfenson, chlorfenvinphos, chlormephos, chlorobenzilate, chloropropylate, chlorothalonil, chlorpropham, chlorpyrifos, chlorpyrifos-methyl, chlorthal-dimethyl, chlorthion, chlorthiophos, epn, epoxiconazole, esfenvalerate, etaconazole, ethalfluralin, ethion, ethofumesate, clodinafop propargyl, clomazone, coumaphos, crimidine, cyanazine, cyanophenphos, cyanophos, cycloate, cyfluthrin, cypermethrin, cyproconazole, cyprodinil, ddd-o,p', ddd-p,p’, dde-o,p’, dde-p,p’, ddm, ddt-o,p', ddt-p,p', deltamethrin, demeton-s, desmetryn, dialifos, diazinon, dichlobenil, dichlofenthion, ditalimfos, heptenophos, hexachlorocyclohexane (hch), alpha, hexachlorocyclohexane (hch), beta, hexaconazole, imazalil, iodofenphos, iprobenfos, iprodione, isocarbophos, isofenphos, isofenphos-methyl, dmst, dodemorph, edifenphos, endosulfan alpha, endosulfan beta, endosulfan sulphate, dichlofluanid, dichloroaniline, 3,5-, dichlorobenzophenone-p,p, dichlorvos, diclobutrazol, dicloran, flusilazole, dicofol, diethofencarb, difenoconazole, dimethachlor, dimethoate, dimethomorph, dimoxystrobin, diniconazole, dioxabenzofos, dioxacarb, dioxathion, diphenylamine, fluchloralin, flucythrinate, fludioxonil, flumetralin, fluorodifen, fluotrimazole, fluquinconazole, flutriafol, folpet, fonofos, formothion, furalaxyl, furathiocarb, gamma-cyhalothrin, halfenprox, kresoxim-methyl, lambda-cyhalothrin, lindane, malaoxon, malathion, mecarbam, mepanipyrim, mepronil, metalaxyl, metazachlor, metconazole, methacrifos, methidathion, methoxychlor, metolachlor, metribuzin, mevinphos, myclobutanil, nitralin, nitrapyrin, nitrothal isopropyl, nuarimol, oxadixyl, oxyfluorfen, paclobutrazol, parathion, parathion-methyl, penconazole, pencycuron, pendimethalin, permethrin, perthane (ethylan), pethoxamid, phenthoate, phorate, phorate sulfoxide, phosalone, phosmet, phosphamidon, phthalimide, picolinafen, picoxystrobin, piperonyl butoxide, piperophos, pirimicarb, pirimicarb desmethyl, pirimiphos-methyl, procymidone, profenofos, profluralin, prometon, prometryn, propachlor, propargite, propazine, propetamphos, propham, propiconazole, propyzamide, prothioconazole destio, prothiofos, pyraclostrobin, pyrazophos, pyridaben, pyrifenox, pyrimethanil, pyriproxyfen, pyroquilon, quinalphos, quinoxyfen, quintozene, resmethrin, spiromesifen, sulfotep, tau-fluvalinate, tebuconazole, tebufenpyrad, tecnazene, tefluthrin, terbacil, terbutryn, tetrachlorvinphos, tetraconazole, tetradifon, tetrahydrophthalimide, tetramethrin, tetrasul, tolclofos-methyl, tolylfluanid, triadimefon, triadimenol, tri-allate, triazophos, trifloxystrobin, triflumizole, trifluralin, vinclozolin,	0.005
	aldrin, dieldrin, disulfoton, fipronil, heptachlor, hexachlorobenzene, nitrofen, terbufos	0.001
	endrin, fipronil desulfinyl, fipronil sulfon, heptachlor cis-epoxid, heptachlor trans-epoxid	0.0025
GC-MS	dithiocarbamates expressed as CS_2 (ferbam, mancozeb, maneb, metam, metiram, nabam, propineb, thiram, ziram, zineb)	0.005
	ethylene oxide (2-chloroethanol expressed as ethylene oxide)	0.02
LC-MS/MS	bromide ion	0.2
	AMPA, maleic hydrazide	0.05
	abamectin, acephate, aclonifen, aldicarb, aldicarb sulfone, aldicarb sulfoxide, amisulbrom, azadirachtin, aziprotryne, bac c10, bac c12, bac c14, bac c8, beflubutamid, bendiocarb, benthiavalicarb isopropyl, bixafen, bromacil, bromuconazole, carbetamide, carbofuran 3-keto, carfentrazone-ethyl, chlorpyrifos, chromafenozide, clodinafop propargyl, clothianidin, cyantraniliprole, cycloxydim, cymiazol, cyproconazole, ddac c8, deet, desmedipham, dicrotophos, diflufenican, dinotefuran, disulfoton, diuron, emamectin b1a, emamectin b1b, ethiofencarb, ethirimol, famoxadone, fenfuram, fenhexamid, fenobucarb, fenpropidin, fenthion, fenthion oxon, fenthion oxon sulphone, fenthion sulfoxide, fenthion sulphone, florasulam, flupyradifurone, flurochloridone, flutianil, flutriafol, fluxapyroxad, formetanate, fosthiazate, halofenozide, imazalil, imazapic, imidacloprid, iodosulfuron methyl, ipconazole, isofetamid, isoprocarb, isoprothiolane, lenacil, lufenuron, malathion, metaflumizone, metamitron, metazachlor, methamidophos, methiocarb sulphone, methomyl, methoprotryne, metobromuron, metosulam, metoxuron, monuron, nicotine, nitenpyram, oxycarboxin, parathion, parathion-methyl, penflufen, penthiopyrad, pethoxamid, phenmedipham, phosmet oxon, phoxim, piperonyl butoxide, prochloraz bts 44595, prochloraz bts 44596, propoxur, propoxycarbazone, pyridafol, pyrifenox, pyriproxyfen, pyroquilon, quinclorac, quinoclamine, rimsulfuron, rotenone, spinetoram c42, spinetoram c43, sulfosulfuron, sulfoxaflor, tebuconazole, teflubenzuron, tepraloxydim, terbufos, terbufos oxon, terbufos sulphone, thifensulfuron-methyl, thiometon, tolfenpyrad, topramezone, tralkoxydim, trichlorfon, tricyclazole, tridemorph, triflumizole, triflumuron, triflusulfuron methyl, triticonazole, tritosulfuron, 2,4,5-t,2,4-d,2,4-db, acibenzolar acid, acifluorfen, aminopyralid, bentazone, bromacil, bromoxynil, clopyralid, dicamba, dichlorprop, diclofop, diflufenzopyr, fenoprop, fenoxaprop-p, fluazifop, fluroxypyr, glyphosate,hal	0.01

	oxyfop, imazamox, imazapyr, imazethapyr, ioxynil, mcpa, mcpb, mecoprop, picloram, quinmerac, quizalofop, triasulfuron, tribenuron methyl, triclopyr, trimethylsulfonyl cation, trinexapac, ethephon chlorate, perchlorates, phosetyl, phosphonic acid, cyromazine, matrine, oxymatrine, melamine, ETU	
	acetamiprid, ametoctradin, amidosulfuron, azoxystrobin, benzovindiflupyr, boscalid, carbaryl, carbendazim, carbofuran, carbofuran 3-hydroxy, chlorantraniliprole, chloridazon, chlorotoluron, chlorsulfuron, clofentezine, cyflufenamid, cyflumetofen, cymoxanil, diethofencarb, diflubenzuron, dimethenamid, dimethoate, disulfoton sulfon, disulfoton sulfoxide, dmf, dmpf, ethametsulfuron-methyl, etoxazole,fenamidone, fenamiphos, fenamiphos sulfoxide, fenamiphos sulphone, fenbuconazole, fenoxaprop-p-ethyl, fenpropimorph, fenpyroximate, flazasulfuron, flonicamid, flufenacet, flufenoxuron, fluopicolide, fluopyram, fluoxastrobin, flutolanil, foramsulfuron, fuberidazole, hexaflumuron, hexythiazox, indoxacarb, iprovalicarb, isoproturon, isopyrazam, isoxaben, isoxaflutole, isoxathion, linuron, malaoxon, mandipropamid,metalaxyl,methiocarb,methiocarb sulphoxide, methoxyfenozide,metolachlor-s,metrafenone, metsulfuron-methyl, monocrotophos, napropamide, nicosulfuron,novaluron,oxadixyl,oxamyl,paraoxon-methyl,pencycuron,pendimethalin,phenthoate, phosmet, pinoxaden,prochloraz,propamocarb, propaquizafop, proquinazid, prosulfocarb, prosulfuron, pyridaben, pyroxsulam, quizalofop-ethyl, saflufenacil, silthiofam, spinosyn a, spinosyn d, spirodiclofen, spirotetramat, spirotetramat enol, spirotetramat enol-glucoside, spirotetramat ketohydroxy,spirotetramat monohydroxy, spiroxamine, sulfometuron methyl, tebufenozide, tebufenpyrad, terbufos sulphoxide, terbuthylazine, thiabendazole, thiacloprid, thiamethoxam, thiencarbazone-methyl, thiodicarb, thiophanate-methyl, zoxamide, cyhexatin, dithianon,dodine,fenbutatin oxide, flonicamid,TFNA,TFNA-AM,TFNG, chlormequat chloride, mepiquat chloride, PTU	0.005
	cadusafos,demeton-s-methyl,demeton-s-methylsulphone,demeton-s-methyl sulphoxide, fensulfothion, fensulfothion oxon, fensulfothion oxon sulphone, fensulfothion sulphone, omethoate, fentin, glufosinate together with matabolites (MPP, glufosinate metabolite, N-acetyl-glufosinate)	0.0025

Table S4. The list of the compounds and corresponding analytical method.

Multiresidue (499 substances)	2-Phenylphenol, Abamectin, Acephate, Acetamiprid, Acetochlor, Aclonifen, Acrinathrin, Alachlor, Aldicarb, Aldicarb sulfone, Aldicarb sulfoxide, Aldrin, Allethrin, Ametoctradin, Ametryn, Amidosulfuron, Aminocarb, Amisulbrom, Anthraquinone, Atrazine, Azaconazole, Azadirachtin, Azinphos ethyl, Azinphos-methyl, Aziprotryne, Azoxystrobin, Beflubutamid, Benalaxyl, Bendiocarb, Benfluralin, Benfuracarb, Benthiavalicarb isopropyl, Benzovindiflupyr, Bifenazate, Bifenazate diazene, Bifenox, Bifenthrin, Biphenyl, Bitertanol, Bixafen, Boscalid, Bromacil, Bromfenvinphos, Bromocyclen, Bromophos, Bromophos-ethyl, Bromopropylate, Bromuconazole, BTS 44595, BTS 44596, Bupirimate, Buprofezin, Butachlor, Butafenacil, Butylate, Cadusafos, Captafol, Captan, Carbaryl, Carbendazim, Carbetamide, Carbofuran, Carbofuran 3-hydroxy, Carbofuran 3-keto, Carboxin, Carfentrazone-ethyl, Chinomethionat, Chlorantraniliprole, Chlorbenside, Chlorbufam, Chlordane,-cis, Chlordane,-oxy, Chlordane,-trans, Chlorfenapyr, Chlorfenson, Chlorfenvinphos, Chloridazon, Chlormephos, Chlorobenzilate, Chloropropylate, Chlorothalonil, Chlorotoluron, Chlorpropham, Chlorpyrifos, Chlorpyrifos-methyl, Chlorsulfuron, Chlorthal-dimethyl, Chlorthiophos, Chlothion, Chromafenozide, Clodinafop propargyl, Clofentezine, Clomazone, Clothianidin, Coumaphos, Crimidine, Cyanazine, cyanophenphos, cyanophos, Cyantraniliprole, Cyazofamid, Cycloate, Cycloxydim, Cyflufenamid, Cyflumetofen, Cyfluthrin, Cymiazol, Cymoxanil, Cypermethrin, Cyprazine, Cyproconazole, Cyprodinil, DDD-o,p', DDD-p,p', DDE-o,p', DDE-p,p', DDM, DDT-o,p', DDT-p,p', DEET, Deltamethrin, Demeton-S, Demeton-S-methyl, Demeton-S-methyl sulphone, Demeton-S-methyl sulphoxide, Desmedipham, Desmetryn, Dialifos, Diazinon, Dichlobenil, Dichlofenthion, Dichlofluanid, Dichloroaniline 3,5-, Dichlorobenzamide 2,6-, Dichlorobenzophenone-p,p, Dichlorvos, Diclobutrazol, Dicloran, Dicofol, Dicrotophos, Dieldrin, Diethofencarb, Difenoconazole, Diflubenzuron, Diflufenican, Dimethachlor, Dimethenamid, Dimethoate, Dimethomorph, Dimoxystrobin, Diniconazole, Dinitramine, Dinobuton, Dinoseb, Dinotefuran, Dioxabenzofos, Dioxacarb, Dioxathion, Diphenylamine, Disulfoton, Disulfoton sulfon, Disulfoton sulfoxide, Ditalimfos, Diuron, DMF, DMPF, DMST, Dodemorph, Edifenphos, Emamectin B1a, Emamectin B1b, Endosulfan alpha, Endosulfan beta, Endosulfan sulphate, Endrin, Endrin keton, EPN, Epoxiconazole, Esfenvalerate, Etaconazole, Ethalfluralin, Ethametsulfuron-methyl, Ethiofencarb, Ethion, Ethirimol, Ethofumesate, Ethofumesate -2-keto, Ethoprophos, Ethoxyquin, Ethylan, Etofenprox, Etoxazole, Etrimfos, Famoxadone, Fenamidone, Fenamiphos, Fenamiphos sulfoxide, Fenamiphos sulphone, Fenarimol, Fenazaquin, Fenbuconazole, Fenchlorphos, Fenfuram, Fenhexamid, Fenitrothion, Fenobucarb, Fenoxaprop-P-ethyl, Fenoxycarb, Fenpropathrin, Fenpropidin, Fenpropimorph, Fenpyrazamine, Fenpyroximate, Fensulfothion, Fensulfothion oxon, Fensulfothion oxon sulphone, Fensulfothion sulphone, Fenthion, Fenthion oxon, Fenthion oxon sulphone, Fenthion sulfoxide, Fenthion sulphone, Fenvalerate, Fipronil, Fipronil desulfinyl, Fipronil sulfon, Flazasulfuron, Flonicamid, Florasulam, Fluchloralin, Flucythrinate, Fludioxonil, Fluensulfone, Flufenacet, Flufenoxuron, Flumetralin, Flumioxazin, Fluopicolide, Fluopyram, Fluorodifen, Fluotrimazole, Fluoxastrobin, Flupyradifurone, Fluquinconazole, Flurochloridone, Flurprimidol, Flurtamone, Flusilazole, Flutianil, Flutolanil, Flutriafol, Fluxapyroxad, Folpet, Fonofos, Foramsulfuron, Formetanate, Formothion, Fosthiazate, Fuberidazole, Furalaxyl, Furathiocarb, Gamma-cyhalothrin, Halfenprox, Halofenozide, Heptachlor, Heptachlor cis-epoxid isomer B, Heptachlor trans-epoxid isomer A, Heptenophos, Hexachlorobenzene, Hexachlorocyclohexane HCH alpha, Hexachlorocyclohexane HCH beta, Hexaconazole, Hexaflumuron, Hexythiazox, Imazalil, Imazapic, Imidacloprid, Indoxacarb, Iodofenphos, Iodosulfuron methyl, Ipconazole, Iprobenfos, Iprodione,

	Iprovalicarb, Isocarbophos, Isofenphos, Isofenphos-methyl, Isofetamid, Isoprocarb, Isoprothiolane, Isoproturon, Isopyrazam, Isoxaben, Isoxaflutole, Isoxathion, Kresoxim-methyl, lambda-Cyhalothrin, Lenacil, Lindane, Linuron, Lufenuron, Malaoxon, Malathion, Mandestrobin, Mandipropamid, Mecarbam, Mepanipyrim, Mepronil, Metaflumizone, Metalaxyl, Metamitron, Metazachlor, Metconazole, Methacrifos, Methamidophos, Methidathion, Methiocarb, Methiocarb sulphone, Methiocarb sulphoxide, Methomyl, Methoprotryne, Methoxychlor, Methoxyfenozide, Metobromuron, Metolachlor, Metolachlor-S, Metosulam, Metoxuron, Metrafenone, Metribuzin, Metsulfuron-methyl, Mevinphos, Molinate, Monocrotophos, Monuron, Myclobutanil, Napropamide, Nicosulfuron, Nitenpyram, Nitralin, Nitrapyrin, Nitrofen, Nitrothal isopropyl, Novaluron, Nuarimol, Omethoate, Oxadiazon, Oxadixyl, Oxamyl, Oxycarboxin, Oxyfluorfen, Paclobutrazol, Paraoxon-methyl, Parathion, Parathion-methyl, Penconazole, Pencycuron, Pendimethalin, Penflufen, Pentachloroaniline, Penthiopyrad, Permethrin, Pethoxamid, Phenmedipham, Phenthoate, Phorate, Phorate sulfone, Phorate sulfoxide, Phosalone, Phosmet, Phosmet oxon, Phosphamidon, Phoxim, Phthalimide, Picolinafen, Picoxystrobin, Pinoxaden, Piperonyl butoxide, Piperophos, Pirimicarb, Pirimicarb desmethyl, Pirimiphos-ethyl, Pirimiphos-methyl, Prochloraz, Procymidone, Profenofos, Profluralin, Prometon, Prometryn, Propachlor, Propamocarb, Propaquizafop, Propargite, Propazine, Propetamphos, Propham, Propiconazole, Propoxur, Propoxycarbazone, Propyzamide, Proquinazid, Prosulfocarb, Prosulfuron, Prothioconazole destio, Prothiofos, Pymetrozine, Pyraclostrobin, Pyrazophos, Pyrethrins, Pyridaben, Pyridafol, Pyridalyl, Pyridaphenthion, Pyrifenox, Pyrimethanil, Pyriofenone, Pyriproxyfen, Pyroquilon, Pyroxsulam, Quinalphos, Quinclorac, Quinoclamine, Quinoxyfen, Quintozene, Quizalofop-ethyl, Resmethrin, Rimsulfuron, Rotenone, Saflufenacil, Silafluofen, Silthiofam, Simazine, Spinetoram C42, Spinetoram C43, Spinosyn A, Spinosyn D, Spirodiclofen, Spiromesifen, Spirotetramat, Spirotetramat enol, Spirotetramat enol-glucoside, Spirotetramat ketohydroxy, Spirotetramat monohydroxy, Spiroxamine, Sulfometuron methyl, Sulfosulfuron, Sulfotep, Sulfoxaflor, tau-Fluvalinate, Tebuconazole, Tebufenozide, Tebufenpyrad, Tecnazene, Teflubenzuron, Tefluthrin, Tepraloxydim, Terbacil, Terbufos, Terbufos oxon, Terbufos sulphone, Terbufos sulphoxide, Terbuthylazine, Terbutryn, Tetrachlorvinphos, Tetraconazole, Tetradifon, Tetrahydrophthalimide, Tetramethrin, Tetrasul, Thiabendazole, Thiacloprid, Thiamethoxam, Thiencarbazone-methyl, Thifensulfuron-methyl, Thiobencarb, Thiodicarb, Thiometon, Thiophanate-methyl, Tolclofos-methyl, Tolfenpyrad, Tolylfluanid, Topramezone, Tralkoxydim, Triadimefon, Triadimenol, Tri-allate, Triazophos, Trichlorfon, Tricyclazole, Tridemorph, Trifloxystrobin, Triflumizole, Triflumuron, Trifluralin, Triflusulfuron, Triticonazole, Tritosulfuron, Vinclozolin, Zoxamide.
Sub-method 1<br>(9 substances)	Cyhexatin, Dithianon, Dodine, Fenbutatin oxide, Fentin, Flonicamid (and its metabolites: TFNA, TFNA-AM, TFNG).
Sub-method 2<br>(33 substances)	2,4,5-T, 2,4-D, 2,4-DB, Acibenzolar acid, Acifluorfen, Aminopyralid, Bentazone, Bromacil, Bromoxynil, Clopyralid, Dicamba, Dichlorprop, Diclofop, Diflufenzopyr, Fenoprop, Fenoxaprop-P, Fluazifop, Fluroxypyr, Haloxyfop, Imazamox, Imazapyr, Imazethapyr, Ioxynil, MCPA, MCPB, Mecoprop, Picloram, Quinmerac, Quizalofop, Triasulfuron, Tribenuron methyl, Triclopyr, Trinexapac.
Dithiocarbamates	Ferbam,Mancozeb, Maneb, Metam, Metiram, Nabam, Propineb, Thiram, Zineb, Ziram
QuPPe-PO	Chlormequat, Cyromazine, Ethephon, Fosetyl-Al, Glufosinate (and its metabolites MPP, NAG), Glyphosate, Maleic hydrazide, Mepiquat, Phosphonic acid, Trimethyl-sulphonium cation,

Table S5. The list of the analytical methods details.

No.	Substance name	LOD (mg/kg)	LOQ (mg/kg)	Working Range (mg/kg)	Recoveries (%)	RSD (%)	Measurement Uncertainty (%)
**MULTI GC METHOD**							
1.	2-Phenylphenol	0,001	0,005	(0.005 - 5)	97	12	34,5
2.	Acetochlor	0,001	0,005	(0.005 - 5)	97	8,3	26,8

3.	Acrinathrin	0,001	0,005	(0.005 - 5)	90	9,95	35,7
4.	Alachlor	0,001	0,005	(0.005 - 5)	99	10	29,6
5.	Aldrin	0,0005	0,001	(0.001 - 5)	98	6,5	21,4
6.	Allethrin	0,001	0,005	(0.005 - 5)	108	8,8	31,5
7.	Ametryn	0,001	0,005	(0.005 - 5)	95	7,6	24,7
8.	Aminocarb	0,001	0,005	(0.005 - 5)	83	12	48,6
9.	Anthraquinone	0,001	0,005	(0.005 - 5)	98	9,8	33,0
10.	Atrazine	0,001	0,005	(0.005 - 5)	101	9,7	27,8
11.	Azaconazole	0,001	0,005	(0.005 - 5)	92	11	37,2
12.	Azinphos ethyl	0,001	0,005	(0.005 - 5)	92	5,5	26,7
13.	Azinphos-methyl	0,001	0,005	(0.005 - 5)	97	12	38,9
14.	Azoxystrobin	0,001	0,005	(0.005 - 5)	99	13	35,8
15.	Beflubutamid	0,001	0,005	(0.005 - 5)	88	5,5	28,1
16.	Benalaxyl	0,001	0,005	(0.005 - 5)	102	6,6	19,5
17.	Benfluralin	0,001	0,005	(0.005 - 5)	88	4,5	32,2
18.	Benfuracarb	0,001	0,005	(0.005 - 5)	93	6,7	32,8
19.	Bifenazate	0,001	0,005	(0.005 - 5)	93	9,7	36,7
20.	Bifenazate diazene	0,001	0,005	(0.005 - 5)	86	9,1	26,0
21.	Bifenox	0,001	0,005	(0.005 - 5)	110	15	46,4
22.	Bifenthrin	0,001	0,005	(0.005 - 5)	98	5,2	18,5
23.	Biphenyl	0,001	0,005	(0.005 - 5)	92	5,7	30,5
24.	Bitertanol	0,001	0,005	(0.005 - 5)	96	5,2	18,0
25.	Boscalid	0,001	0,005	(0.005 - 5)	103	6,4	22,0
26.	Bromfenvinphos	0,001	0,005	(0.005 - 5)	97	7,9	26,3
27.	Bromocyclen	0,001	0,005	(0.005 - 5)	98	7,8	24,4
28.	Bromophos	0,001	0,005	(0.005 - 5)	105	8,8	26,8
29.	Bromophos-ethyl	0,001	0,005	(0.005 - 5)	97	5,00	19,7
30.	Bromopropylate	0,001	0,005	(0.005 - 5)	101	7,5	21,4
31.	Bupirimate	0,001	0,005	(0.005 - 5)	100	5,2	17,7

32.	Buprofezin	0,001	0,005	(0.005 - 5)	102	10	29,9
33.	Butachlor	0,001	0,005	(0.005 - 5)	98	12	38,9
34.	Butafenacil	0,001	0,005	(0.005 - 5)	107	15	45,3
35.	Butylate	0,001	0,005	(0.005 - 5)	97	12	34,5
36.	Captafol	0,001	0,005	(0.005 - 5)	101	8,4	27,0
37.	Captan	0,001	0,005	(0.005 - 5)	101	9,8	31,4
38.	Carbaryl	0,001	0,005	(0.005 - 5)	91	14	43,3
39.	Carboxin	0,001	0,005	(0.005 - 5)	102	13	39,8
40.	Chinomethionat	0,001	0,005	(0.005 - 5)	91	9,9	37,0
41.	Chlorbenside	0,001	0,005	(0.005 - 5)	98	6,7	29,1
42.	Chlorbufam	0,001	0,005	(0.005 - 5)	101	13,3	38,5
43.	Chlordane, -cis	0,001	0,005	(0.005 - 5)	95	11	31,0
44.	Chlordane, -oxy	0,002	0,01	(0.01 - 5)	98	5,4	15,0
45.	Chlordane, -trans	0,002	0,01	(0.01 - 5)	97	11	29,0
46.	Chlorfenapyr	0,001	0,005	(0.005 - 5)	102	4,5	22,9
47.	Chlorfenson	0,001	0,005	(0.005 - 5)	99	5,7	17,0
48.	Chlorfenvinphos	0,001	0,005	(0.005 - 5)	99	9,5	29,9
49.	Chlormephos	0,001	0,005	(0.005 - 5)	95,5	12	34,9
50.	Chlorobenzilate	0,001	0,005	(0.005 - 5)	100	5	14,7
51.	Chloropropylate	0,001	0,005	(0.005 - 5)	99	5,4	19,8
52.	Chlorothalonil	0,001	0,005	(0.005 - 5)	103	8,7	27,5
53.	Chlorpropham	0,001	0,005	(0.005 - 5)	108	6,3	23,8
54.	Chlorpyrifos	0,001	0,005	(0.005 - 5)	98	8	23,8
55.	Chlorpyrifos-methyl	0,001	0,005	(0.005 - 5)	103	6,1	18,1
56.	Chlorthal-dimethyl	0,001	0,005	(0.005 - 5)	99	3,8	12,5
57.	Chlorthion	0,001	0,005	(0.005 - 5)	92	9,8	36,9
58.	Chlorthiophos	0,001	0,005	(0.005 - 5)	96	7,8	27,7
59.	Clodinafop propargyl	0,001	0,005	(0.005 - 5)	89	7,4	42,1
60.	Clomazone	0,001	0,005	(0.005 - 5)	104	8,7	25,9

61.	Coumaphos	0,001	0,005	(0.005 - 5)	99	11	43,6
62.	Crimidine	0,001	0,005	(0.005 - 5)	103	12	34,6
63.	Cyanazine	0,001	0,005	(0.005 - 5)	105	8,2	30,4
64.	Cyanophenphos	0,001	0,005	(0.005 - 5)	105	3,9	17,8
65.	Cyanophos	0,001	0,005	(0.005 - 5)	98	12,6	41,7
66.	Cycloate	0,001	0,005	(0.005 - 5)	105	9	32,7
67.	Cyfluthrin	0,001	0,005	(0.005 - 5)	99	9,9	29,1
68.	Cypermethrin	0,001	0,005	(0.005 - 5)	96	11	31,4
69.	Cyprazine	0,002	0,01	(0.01 - 5)	103	12	23,9
70.	Cyproconazole	0,001	0,005	(0.005 - 5)	96	7,2	39,4
71.	Cyprodinil	0,001	0,005	(0.005 - 5)	109	7,3	26,6
72.	DDD-o,p'	0,001	0,005	(0.005 - 5)	107	5,5	20,8
73.	DDD-p,p'	0,001	0,005	(0.005 - 5)	109	4,6	23,0
74.	DDE-o,p'	0,001	0,005	(0.005 - 5)	94	5,7	19,9
75.	DDE-p,p'	0,001	0,005	(0.005 - 5)	108	4,4	19,6
76.	DDM	0,001	0,005	(0.005 - 5)	99	3,8	12,5
77.	DDT-o,p'	0,001	0,005	(0.005 - 5)	88	8,2	33,7
78.	DDT-p,p'	0,001	0,005	(0.005 - 5)	92	6,1	24,0
79.	Deltamethrin	0,001	0,005	(0.005 - 5)	90	9,7	34,4
80.	Demeton-S	0,001	0,005	(0.005 - 5)	96	6,6	27,8
81.	Desmetryn	0,001	0,005	(0.005 - 5)	102	10,3	32,9
82.	Dialifos	0,001	0,005	(0.005 - 5)	99	5,5	21,7
83.	Diazinon	0,001	0,005	(0.005 - 5)	99	6	18,2
84.	Dichlobenil	0,001	0,005	(0.005 - 5)	108	4,8	20,3
85.	Dichlofenthion	0,001	0,005	(0.005 - 5)	109	10	33,7
86.	Dichlofluanid	0,001	0,005	(0.005 - 5)	88	9,7	36,5
87.	Dichloroaniline, 3,5-	0,001	0,005	(0.005 - 5)	99	16	47,9
88.	Dichlorobenzamide, 2,6-	0,002	0,01	(0.01 - 5)	90	11	22,9
89.	Dichlorobenzophenone-p,p	0,001	0,005	(0.005 - 5)	96	3,8	17,4

90.	Dichlorvos	0,001	0,005	(0.005 - 5)	98	6,7	21,8
91.	Diclobutrazol	0,001	0,005	(0.005 - 5)	92	11	34,0
92.	Dicloran	0,001	0,005	(0.005 - 5)	97	12	36,4
93.	Dicofol	0,001	0,005	(0.005 - 5)	102	6,4	20,7
94.	Dieldrin	0,0005	0,001	(0.001 - 5)	100	16	45,9
95.	Diethofencarb	0,001	0,005	(0.005 - 5)	99	10	36,2
96.	Difenoconazole	0,001	0,005	(0.005 - 5)	93	16	48,3
97.	Dimethachlor	0,001	0,005	(0.005 - 5)	100	5,3	17,8
98.	Dimethoate	0,001	0,005	(0.005 - 5)	97	7,5	27,2
99.	Dimethomorph	0,001	0,005	(0.005 - 5)	95	8,6	29,0
100.	Dimoxystrobin	0,001	0,005	(0.005 - 5)	97	4,2	33,2
101.	Diniconazole	0,001	0,005	(0.005 - 5)	91	8	33,5
102.	Dinitramine	0,002	0,01	(0.01 - 5)	83	5	48,6
103.	Dinobuton	0,002	0,01	(0.01 - 5)	92	14	44,4
104.	Dinoseb	0,002	0,01	(0.01 - 5)	90	7,9	22,0
105.	Dioxabenzofos	0,001	0,005	(0.005 - 5)	98	14	40,7
106.	Dioxacarb	0,001	0,005	(0.005 - 5)	86	6,8	34,0
107.	Dioxathion	0,001	0,005	(0.005 - 5)	96	12	37,4
108.	Diphenylamine	0,001	0,005	(0.005 - 5)	104	5	22,8
109.	Disulfoton	0,0005	0,001	(0.001 - 5)	98	7	26,4
110.	Disulfoton sulfon	0,002	0,01	(0.01 - 5)	88	5,4	15,0
111.	Disulfoton sulfoxide	0,002	0,01	(0.01 - 5)	75	11	30,0
112.	Ditalimfos	0,001	0,005	(0.005 - 5)	93	9,2	36,0
113.	DMST	0,001	0,005	(0.005 - 5)	104	8	24,0
114.	Dodemorph	0,001	0,005	(0.005 - 5)	93	9,3	34,4
115.	Edifenphos	0,001	0,005	(0.005 - 5)	104	9,7	28,2
116.	Endosulfan alpha	0,001	0,005	(0.005 - 5)	110	9,2	32,6
117.	Endosulfan beta	0,001	0,005	(0.005 - 5)	105	5,5	31,4
118.	Endosulfan sulphate	0,001	0,005	(0.005 - 5)	96	3,8	15,0

119.	Endrin	0,001	0,0025	(0.0025 - 5)	93	6,9	28,0
120.	Endrin keton	0,002	0,01	(0.01 - 5)	87	3,2	9,0
121.	EPN	0,001	0,005	(0.005 - 5)	109	5,2	22,8
122.	Epoxiconazole	0,001	0,005	(0.005 - 5)	97	5,2	33,1
123.	Esfenvalerate	0,001	0,005	(0.005 - 5)	94	10	39,9
124.	Etaconazole	0,001	0,005	(0.005 - 5)	99	8,1	26,8
125.	Ethalfluralin	0,001	0,005	(0.005 - 5)	87	5,2	35,0
126.	Ethion	0,001	0,005	(0.005 - 5)	97	3,3	27,0
127.	Ethofumesate	0,001	0,005	(0.005 - 5)	105	7,3	23,0
128.	Ethofumesate, -2-keto	0,002	0,01	(0.01 - 5)	80	4	11,0
129.	Ethoprophos	0,001	0,005	(0.005 - 5)	97	4,3	14,2
130.	Ethoxyquin	0,001	0,005	(0.005 - 5)	89	7,8	32,0
131.	Etofenprox	0,001	0,005	(0.005 - 5)	83	7,4	40,6
132.	Etrimfos	0,001	0,005	(0.005 - 5)	101	4,9	15,5
133.	Fenamiphos	0,001	0,005	(0.005 - 5)	103	11	33,1
134.	Fenarimol	0,001	0,005	(0.005 - 5)	109	2,2	19,7
135.	Fenazaquin	0,001	0,005	(0.005 - 5)	101	12	33,6
136.	Fenbuconazole	0,001	0,005	(0.005 - 5)	86	9,3	38,8
137.	Fenchlorphos	0,001	0,005	(0.005 - 5)	89	8,9	35,3
138.	Fenhexamid	0,001	0,005	(0.005 - 5)	103	7,1	21,3
139.	Fenitrothion	0,001	0,005	(0.005 - 5)	94	3,2	24,3
140.	Fenoxycarb	0,001	0,005	(0.005 - 5)	95	12	39,4
141.	Fenpropathrin	0,001	0,005	(0.005 - 5)	92	4,9	21,1
142.	Fenpropidin	0,001	0,005	(0.005 - 5)	103	5,7	23,1
143.	Fenpropimorph	0,001	0,005	(0.005 - 5)	88	5,7	29,2
144.	Fenpyrazamine	0,002	0,01	(0.01 - 5	83	9	25,0
145.	Fenthion	0,001	0,005	(0.005 - 5)	95	5,9	19,2
146.	Fenvalerate	0,001	0,005	(0.005 - 5)	94	10	39,9
147.	Fipronil	0,0005	0,001	(0.001 - 5)	106	6,6	22,1

148.	Fipronil desulfinyl	0,001	0,0025	(0.0025 - 5)	92	6,9	27,6
149.	Fipronil sulfon	0,001	0,0025	(0.0025 - 5)	99	13	25,3
150.	Fluchloralin	0,001	0,005	(0.005 - 5)	94	8,7	29,6
151.	Flucythrinate	0,001	0,005	(0.005 - 5)	89	7,6	31,1
152.	Fludioxonil	0,001	0,005	(0.005 - 5)	93	12	45,5
153.	Fluensulfone	0,002	0,01	(0.01 - 5)	81	4,3	12,0
154.	Flumetralin	0,001	0,005	(0.005 - 5)	87	5,9	41,9
155.	Flumioxazin	0,002	0,01	(0.01 - 5)	92	4	13,0
156.	Fluorodifen	0,001	0,005	(0.005 - 5)	97	11	33,7
157.	Fluotrimazole	0,001	0,005	(0.005 - 5)	99	7,7	25,6
158.	Fluquinconazole	0,001	0,005	(0.005 - 5)	91	6,8	26,1
159.	Flurprimidol	0,002	0,01	(0.01 - 5)	89	3,1	8,6
160.	Flurtamone	0,002	0,01	(0.01 - 5)	83	18	36,1
161.	Flusilazole	0,001	0,005	(0.005 - 5)	98	5	21,7
162.	Flutianil	0,002	0,01	(0.01 - 5)	77	3,6	10,0
163.	Flutriafol	0,001	0,005	(0.005 - 5)	98	8	25,0
164.	Folpet	0,001	0,005	(0.005 - 5)	92	13	41,5
165.	Fonofos	0,001	0,005	(0.005 - 5)	107	7,6	25,4
166.	Formothion	0,001	0,005	(0.005 - 5)	102	7,4	21,6
167.	Furalaxyl	0,001	0,005	(0.005 - 5)	104	6,7	20,5
168.	Furathiocarb	0,001	0,005	(0.005 - 5)	104	13	42,7
169.	Gamma-cyhalothrin	0,001	0,005	(0.005 - 5)	107	13	42,9
170.	Halfenprox	0,001	0,005	(0.005 - 5)	90	7,0	45,0
171.	Heptachlor	0,0005	0,001	(0.001 - 5)	87	6,5	31,5
172.	Heptachlor cis-epoxid	0,001	0,0025	(0.0025 - 5)	97	6,3	30,0
173.	Heptachlor trans-epoxid	0,001	0,0025	(0.0025 - 5)	103	9	17,9
174.	Heptenophos	0,001	0,005	(0.005 - 5)	102	9,1	28,0
175.	Hexachlorobenzene	0,0005	0,001	(0.001 - 5)	107	6,2	25,1
176.	Hexachlorocyclohexane (HCH), alpha	0,001	0,005	(0.005 - 5)	106	5,3	19,7

177.	Hexachlorocyclohexane (HCH), beta	0,001	0,005	(0.005 - 5)	105	7,8	24,3
178.	Hexaconazole	0,001	0,005	(0.005 - 5)	99	9,3	31,5
179.	Imazalil	0,001	0,005	(0.005 - 5)	101	13	39,3
180.	Iodofenphos	0,001	0,005	(0.005 - 5)	97	8,2	25,4
181.	Iprobenfos	0,001	0,005	(0.005 - 5)	101	11	31,9
182.	Iprodione	0,001	0,005	(0.005 - 5)	94	6,8	24,9
183.	Isocarbophos	0,001	0,005	(0.005 - 5)	109	8,8	30,3
184.	Isofenphos	0,001	0,005	(0.005 - 5)	104	6,7	21,0
185.	Isofenphos-methyl	0,001	0,005	(0.005 - 5)	96	6,5	21,1
186.	Isofetamid	0,002	0,01	(0.01 - 5)	76	2,7	7,7
187.	Kresoxim-methyl	0,001	0,005	(0.005 - 5)	104	8,5	25,1
188.	lambda-Cyhalothrin	0,001	0,005	(0.005 - 5)	99	8,4	26,6
189.	Lindane	0,001	0,005	(0.005 - 5)	102	8,1	25,9
190.	Malaoxon	0,001	0,005	(0.005 - 5)	96	6,7	21,6
191.	Malathion	0,001	0,005	(0.005 - 5)	109	5,4	23,4
192.	Mandestrobin	0,002	0,01	(0.01 - 5)	77	4,2	12,0
193.	Mecarbam	0,001	0,005	(0.005 - 5)	103	7,6	23,7
194.	Mepanipyrim	0,001	0,005	(0.005 - 5)	94	10	31,9
195.	Mepronil	0,001	0,005	(0.005 - 5)	97	9,3	31,0
196.	Metalaxyl	0,001	0,005	(0.005 - 5)	100	12	36,5
197.	Metazachlor	0,001	0,005	(0.005 - 5)	107	7,1	24,8
198.	Metconazole	0,001	0,005	(0.005 - 5)	106	8	25,8
199.	Methacrifos	0,001	0,005	(0.005 - 5)	110	6,4	26,4
200.	Methidathion	0,001	0,005	(0.005 - 5)	106	5,1	19,3
201.	Methoxychlor	0,001	0,005	(0.005 - 5)	101	11	32,8
202.	Metolachlor	0,001	0,005	(0.005 - 5)	108	4,4	20,5
203.	Metribuzin	0,001	0,005	(0.005 - 5)	110	5,5	25,0
204.	Mevinphos	0,001	0,005	(0.005 - 5)	92	11	34,9
205.	Molinate	0,002	0,01	(0.01 - 5)	94	6,2	17,0

206.	Myclobutanil	0,001	0,005	(0.005 - 5)	99	6,7	23,8
207.	Nitralin	0,001	0,005	(0.005 - 5)	87	5,7	40,3
208.	Nitrapyrin	0,001	0,005	(0.005 - 5)	92	8	27,8
209.	Nitrofen	0,0005	0,001	(0.001 - 5)	87	9,1	36,1
210.	Nitrothal isopropyl	0,001	0,005	(0.005 - 5)	90	7,3	39,2
211.	Nuarimol	0,001	0,005	(0.005 - 5)	92	10	30,2
212.	Oxadiazon	0,002	0,01	(0.01 - 5)	92	3,2	9,0
213.	Oxadixyl	0,001	0,005	(0.005 - 5)	106	4,7	18,2
214.	Oxyfluorfen	0,001	0,005	(0.005 - 5)	101	8	24,9
215.	Paclobutrazol	0,001	0,005	(0.005 - 5)	85	6,7	35,8
216.	Parathion	0,001	0,005	(0.005 - 5)	101	11	30,3
217.	Parathion-methyl	0,001	0,005	(0.005 - 5)	101	6,5	20,6
218.	Penconazole	0,001	0,005	(0.005 - 5)	104	6,2	19,3
219.	Pencycuron	0,001	0,005	(0.005 - 5)	99	7,7	24,1
220.	Pendimethalin	0,001	0,005	(0.005 - 5)	103	11	32,2
221.	Penflufen	0,002	0,01	(0.01 - 5)	91	4	11,0
222.	Pentachloroaniline	0,002	0,01	(0.01 - 5)	96	11	31,0
223.	Permethrin	0,001	0,005	(0.005 - 5)	89	8,5	32,9
224.	Perthane (Ethylan)	0,001	0,005	(0.005 - 5)	100	3,6	15,7
225.	Pethoxamid	0,001	0,005	(0.005 - 5)	89	3	10,0
226.	Phenthoate	0,001	0,005	(0.005 - 5)	97	3,8	21,1
227.	Phorate	0,001	0,005	(0.005 - 5)	103	6,3	22,2
228.	Phorate sulfone	0,002	0,01	(0.01 - 5)	90	16	32,8
229.	Phorate sulfoxide	0,001	0,005	(0.005 - 5)	102	7,7	23,2
230.	Phosalone	0,001	0,005	(0.005 - 5)	89	6,8	29,0
231.	Phosmet	0,001	0,005	(0.005 - 5)	84	4,6	34,8
232.	Phosphamidon	0,001	0,005	(0.005 - 5)	91	10	34,0
233.	Phthalimide	0,001	0,005	(0.005 - 5)	100	7,8	22,7
234.	Picolinafen	0,001	0,005	(0.005 - 5)	106	8,9	32,8

235.	Picoxystrobin	0,001	0,005	(0.005 - 5)	107	9,7	30,7
236.	Piperonyl butoxide	0,001	0,005	(0.005 - 5)	101	14	41,0
237.	Piperophos	0,001	0,005	(0.005 - 5)	94	7,9	33,7
238.	Pirimicarb	0,001	0,005	(0.005 - 5)	105	5,7	19,1
239.	Pirimicarb desmethyl	0,001	0,005	(0.005 - 5)	90	7,2	29,2
240.	Pirimiphos-ethyl	0,002	0,01	(0.01 - 5)	83	4	10,0
241.	Pirimiphos-methyl	0,001	0,005	(0.005 - 5)	107	6,4	23,2
242.	Procymidone	0,001	0,005	(0.005 - 5)	101	4,7	16,9
243.	Profenofos	0,001	0,005	(0.005 - 5)	86	6,5	32,7
244.	Profluralin	0,001	0,005	(0.005 - 5)	101	6,2	20,3
245.	Prometon	0,001	0,005	(0.005 - 5)	102	11	34,6
246.	Prometryn	0,001	0,005	(0.005 - 5)	103	6,3	19,4
247.	Propachlor	0,001	0,005	(0.005 - 5)	96	9,5	29,9
248.	Propargite	0,001	0,005	(0.005 - 5)	92	5,5	22,8
249.	Propazine	0,001	0,005	(0.005 - 5)	101	5,3	15,8
250.	Propetamphos	0,001	0,005	(0.005 - 5)	98	10	30,6
251.	Propham	0,001	0,005	(0.005 - 5)	106	6	20,7
252.	Propiconazole	0,001	0,005	(0.005 - 5)	96	12	36,3
253.	Propyzamide	0,001	0,005	(0.005 - 5)	101	3,5	15,6
254.	Prothioconazole destio	0,001	0,005	(0.005 - 5)	94	6,6	22,1
255.	Prothiofos	0,001	0,005	(0.005 - 5)	94	9,2	28,9
256.	Pyraclostrobin	0,001	0,005	(0.005 - 5)	92	12	37,7
257.	Pyrazophos	0,001	0,005	(0.005 - 5)	91	4,4	31,2
258.	Pyrethrins	0,01	0,05	(0.05 - 5)	87	7,1	34,0
259.	Pyridaben	0,001	0,005	(0.005 - 5)	103	6,1	22,3
260.	Pyridalyl	0,002	0,01	(0.01 - 5)	90	8	23,0
261.	Pyridaphenthion	0,002	0,01	(0.01 - 5)	82	7,4	21,0
262.	Pyrifenox	0,001	0,005	(0.005 - 5)	95	8,3	30,8
263.	Pyrimethanil	0,001	0,005	(0.005 - 5)	101	3,6	14,6

264.	Pyriofenone	0,002	0,01	(0.01 - 5)	76	4	11,0
265.	Pyriproxyfen	0,001	0,005	(0.005 - 5)	92	9	30,7
266.	Pyroquilon	0,001	0,005	(0.005 - 5)	97	12	40,5
267.	Quinalphos	0,001	0,005	(0.005 - 5)	105	11	34,9
268.	Quinoxyfen	0,001	0,005	(0.005 - 5)	104	5,9	18,4
269.	Quintozene	0,001	0,005	(0.005 - 5)	105	11	36,1
270.	Resmethrin	0,001	0,005	(0.005 - 5)	91	7,6	29,9
271.	Silafluofen	0,002	0,01	(0.01 - 5)	88	3,5	9,8
272.	Simazine	0,002	0,01	(0.01 - 5)	100	11	33,0
273.	Spiromesifen	0,001	0,005	(0.005 - 5)	101	8,3	24,7
274.	Sulfotep	0,001	0,005	(0.005 - 5)	101	10	30,2
275.	tau-Fluvalinate	0,001	0,005	(0.005 - 5)	95	11	35,5
276.	Tebuconazole	0,001	0,005	(0.005 - 5)	90	11	36,6
277.	Tebufenpyrad	0,001	0,005	(0.005 - 5)	105	4,4	29,0
278.	Tecnazene	0,001	0,005	(0.005 - 5)	104	6,7	20,5
279.	Tefluthrin	0,001	0,005	(0.005 - 5)	101	2,9	12,5
280.	Terbacil	0,001	0,005	(0.005 - 5)	94	5,2	21,4
281.	Terbufos	0,0005	0,001	(0.001 - 5)	104	3,7	15,3
282.	Terbutryn	0,001	0,005	(0.005 - 5)	98	2,8	12,5
283.	Tetrachlorvinphos	0,001	0,005	(0.005 - 5)	88	12	40,7
284.	Tetraconazole	0,001	0,005	(0.005 - 5)	90	7,6	28,9
285.	Tetradifon	0,001	0,005	(0.005 - 5)	97	5	17,9
286.	Tetrahydrophthalimide	0,001	0,005	(0.005 - 5)	102	13	39,7
287.	Tetramethrin	0,001	0,005	(0.005 - 5)	90	5,2	16,5
288.	Tetrasul	0,001	0,005	(0.005 - 5)	96	5,6	18,1
289.	Thiobencarb	0,002	0,01	(0.01 - 5)	95	9,4	18,8
290.	Tolclofos-methyl	0,001	0,005	(0.005 - 5)	101	8,2	23,3
291.	Tolylfluanid	0,001	0,005	(0.005 - 5)	85	7,9	37,9
292.	Triadimefon	0,001	0,005	(0.005 - 5)	97	5,3	26,9

293.	Triadimenol	0,001	0,005	(0.005 - 5)	84	7,6	39,1
294.	Tri-allate	0,001	0,005	(0.005 - 5)	102	5,8	19,0
295.	Triazophos	0,001	0,005	(0.005 - 5)	100	9,8	29,5
296.	Trifloxystrobin	0,001	0,005	(0.005 - 5)	95	4,7	18,6
297.	Triflumizole	0,001	0,005	(0.005 - 5)	92	5,8	26,5
298.	Trifluralin	0,001	0,005	(0.005 - 5)	104	4,4	15,6
299.	Vinclozolin	0,001	0,005	(0.005 - 5)	103	4,5	16,8
**MULTI LC METHOD**							
1.	Avermectin	0,002	0,01	(0.01-2)	93	14,0	44,0
2.	Acephate	0,002	0,01	(0.01-2)	86	7,9	35,8
3.	Acetamiprid	0,001	0,005	(0.005-2)	91	3,0	19,1
4.	Aclonifen	0,0025	0,01	(0.01-1)	104	8,7	34,9
5.	Aldicarb	0,002	0,01	(0.01-2)	97	3,5	11,7
6.	Aldicarb sulfone	0,005	0,01	(0.01-2)	105	3,2	19,3
7.	Aldicarb sulfoxide	0,001	0,01	(0.01-2)	95	2,5	13,1
8.	Ametoctradin	0,001	0,005	(0.005-2)	91	5,5	24,0
9.	Amidosulfuron	0,001	0,005	(0.005-2)	80	3,5	41,3
10.	Amisulbrom	0,0025	0,01	(0.01-1)	100	6,2	21,8
11.	Azadirachtin	0,002	0,01	(0.01-2)	82	13,0	46,0
12.	Aziprotryne	0,0025	0,01	(0.01-1)	102	3,8	17,5
13.	Azoxystrobin	0,001	0,005	(0.005-2)	91	4,7	27,1
14.	BAC C10	0,002	0,01	(0.01-2)	101	5,0	12,0
15.	BAC C12	0,002	0,01	(0.01-2)	99	5,0	15,0
16.	BAC C14	0,002	0,01	(0.01-2)	82	5,0	41,0
17.	BAC C8	0,002	0,01	(0.01-2)	95	3,0	14,0
18.	Beflubutamid	0,0025	0,01	(0.01-1)	103	3,7	16,7
19.	Bendiocarb	0,0025	0,01	(0.01-1)	103	5,3	19,3
20.	Benthiavalicarb isopropyl	0,0025	0,01	(0.01-1)	102	2,5	20,8

21.	Benzovindiflupyr	0,001	0,005	(0.005-2)	110	7,0	27,0
22.	Bixafen	0,0025	0,01	(0.01-1)	105	4,8	29,3
23.	Boscalid	0,002	0,005	(0.005-2)	89	5,2	27,1
24.	Bromacil	0,0025	0,01	(0.01-1)	102	2,5	14,4
25.	Bromuconazole	0,004	0,01	(0.01-2)	97	7,1	21,0
26.	Cadusafos	0,001	0,0025	(0.0025-0.5)	94	3,5	16,2
27.	Carbaryl	0,0005	0,005	(0.005-2)	97	4,9	15,4
28.	Carbendazim	0,001	0,005	(0.005-2)	94	3,8	16,3
29.	Carbetamide	0,0025	0,01	(0.01-1)	101	2,5	15,1
30.	Carbofuran	0,0005	0,005	(0.005-2)	98	2,9	14,9
31.	Carbofuran 3-hydroxy	0,001	0,005	(0.005-2)	103	6,7	19,8
32.	Carbofuran 3-keto	0,001	0,01	(0.01-2)	98	3,0	17,0
33.	Carfentrazone-ethyl	0,002	0,01	(0.01-2)	102	10,0	21,0
34.	Chlorantraniliprole	0,001	0,005	(0.005-2)	96	5,3	18,7
35.	Chloridazon	0,001	0,005	(0.005-2)	97	3,2	15,5
36.	Chlorotoluron	0,001	0,005	(0.005-2)	100	3,7	11,1
37.	Chlorpyrifos	0,0025	0,01	(0.01-1)	103	3,0	23,3
38.	Chlorsulfuron	0,002	0,005	(0.005-2)	76	2,9	48,5
39.	Chromafenozide	0,0025	0,01	(0.01-1)	103	2,2	16,2
40.	Clodinafop propargyl	0,002	0,01	(0.01-2)	96	3,9	16,0
41.	Clofentezine	0,0005	0,005	(0.005-2)	95	3,7	14,1
42.	Clothianidin	0,002	0,01	(0.01-2)	103	4,3	16,4
43.	Cyantraniliprole	0,002	0,01	(0.01-2)	84	16,0	36,0
44.	Cyazofamid	0,001	0,005	(0.005-2)	97	3,3	11,0
45.	Cycloxydim	0,001	0,005	(0.005-2)	96	3,2	11,9
46.	Cyflufenamid	0,001	0,005	(0.005-2)	93	3,6	17,9
47.	Cyflumetofen	0,001	0,005	(0.005-2)	108	7,0	19,0
48.	Cymiazol	0,0025	0,01	(0.01-1)	100	2,9	21,6
49.	Cymoxanil	0,001	0,005	(0.005-2)	98	3,0	9,2

50.	Cyproconazole	0,002	0,01	(0.01-2)	101	3,0	13,3
51.	DDAC C8	0,002	0,01	(0.01-2)	102	5,0	15,0
52.	DEET	0,002	0,01	(0.01-2)	100	7,9	22,7
53.	Demeton-S-methyl	0,001	0,0025	(0.0025-0.5)	93	9,7	17,1
54.	Demeton-S-methyl-sulphone	0,001	0,0025	(0.0025-0.5)	94	3,9	15,8
55.	Demeton-S-methyl-sulphoxide	0,001	0,0025	(0.0025-0.5)	92	7,0	25,5
56.	Desmedipham	0,001	0,01	(0.01-2)	97	3,0	10,6
57.	Dicrotophos	0,0025	0,01	(0.01-1)	100	2,6	13,4
58.	Diethofencarb	0,0005	0,005	(0.005-2)	101	2,0	8,4
59.	Diflubenzuron	0,002	0,005	(0.005-2)	94	7,5	30,2
60.	Diflufenican	0,0025	0,01	(0.01-1)	101	3,3	26,0
61.	Dimethenamid	0,001	0,005	(0.005-2)	100	8,0	17,0
62.	Dimethoate	0,001	0,005	(0.005-2)	97	5,5	17,6
63.	Dinoterfuran	0,002	0,01	(0.01-2)	103	6,0	13,0
64.	Disulfoton	0,002	0,01	(0.01-2)	82	4,1	47,0
65.	Disulfoton sulfon	0,001	0,005	(0.005-2)	96	4,0	14,2
66.	Disulfoton sulfoxide	0,001	0,005	(0.005-2)	96	4,0	15,0
67.	Diuron	0,0025	0,01	(0.01-1)	102	3,1	12,4
68.	DMF	0,0005	0,005	(0.005-2)	100	6,3	18,2
69.	DMPF	0,0005	0,005	(0.005-2)	92	2,9	18,9
70.	Emamectin	0,0025	0,01	(0.01-1)	103	2,8	22,5
71.	Ethametsulfuron-methyl	0,001	0,005	(0.005-2)	96	8,0	20,0
72.	Ethiofencarb	0,0025	0,01	(0.01-1)	101	3,0	12,4
73.	Ethirimol	0,0025	0,01	(0.01-1)	94	2,9	18,6
74.	Etoxazole	0,0005	0,005	(0.005-2)	88	3,6	25,4
75.	Famoxadone	0,0025	0,01	(0.01-1)	100	4,1	30,9
76.	Fenamidone	0,001	0,005	(0.005-2)	94	3,2	15,6
77.	Fenamiphos	0,001	0,005	(0.005-2)	94	3,3	15,9
78.	Fenamiphos sulfoxide	0,0005	0,005	(0.005-2)	96	2,0	10,6

79.	Fenamiphos sulphone	0,001	0,005	(0.005-2)	99	3,0	10,4
80.	Fenbuconazole	0,002	0,005	(0.005-2)	102	10,3	33,7
81.	Fenfuram	0,0025	0,01	(0.01-1)	104	3,0	16,5
82.	Fenhexamid	0,0025	0,01	(0.01-1)	94	5,3	18,9
83.	Fenobucarb	0,0025	0,01	(0.01-1)	102	4,1	15,1
84.	Fenoxaprop-P-ethyl	0,0005	0,005	(0.005-2)	96	2,6	12,8
85.	Fenpropidin	0,0025	0,01	(0.01-1)	102	2,7	16,5
86.	Fenpropimorph	0,001	0,005	(0.005-2)	98	3,8	12,0
87.	Fenpyroximate	0,0005	0,005	(0.005-2)	99	3,7	15,7
88.	Fensulfothion	0,001	0,0025	(0.0025-0.5)	92	4,7	20,3
89.	Fensulfothion oxon	0,001	0,0025	(0.0025-0.5)	92	4	19,6
90.	Fensulfothion oxon sulphone	0,001	0,0025	(0.0025-0.5)	99	5,7	16,4
91.	Fensulfothion sulphone	0,001	0,0025	(0.0025-0.5)	94	4,8	18,7
92.	Fenthion	0,0025	0,01	(0.01-1)	103	9,7	39,5
93.	Fenthion oxon	0,002	0,01	(0.01-2)	92	2,8	17
94.	Fenthion oxon sulphone	0,002	0,01	(0.01-2)	88	2,4	25
95.	Fenthion sulfoxide	0,0025	0,01	(0.01-1)	105	3,2	14,3
96.	Fenthion sulphone	0,002	0,01	(0.01-2)	91	3,5	20,0
97.	Flazasulfuron	0,001	0,005	(0.005-2)	96	8,0	20,0
98.	Flonicamid	0,001	0,005	(0.005-2)	94	2,7	15,1
99.	Florasulam	0,002	0,01	(0.01-2)	84	5,0	43,0
100.	Flufenacet	0,001	0,005	(0.005-2)	99	3,4	10,6
101.	Flufenoxuron	0,001	0,005	(0.005-2)	92	5,9	29,6
102.	Fluopicolide	0,001	0,005	(0.005-2)	99	8,0	16,0
103.	Fluopyram	0,001	0,005	(0.005-2)	101	4,0	11,0
104.	Fluoxastrobin	0,001	0,005	(0.005-2)	100	7,0	22,5
105.	Flupyradifurone	0,002	0,01	(0.01-2)	81	19,0	39,0
106.	Flurochloridone	0,005	0,01	(0.01-2)	96	9,2	27,6
107.	Flutianil	0,002	0,01	(0.01-2)	90	4,6	24,0

108.	Flutolanil	0,001	0,005	(0.005-2)	98	2,0	6,6
109.	Flutriafol	0,001	0,01	(0.01-2)	99	3,0	9,4
110.	Fluxapyroxad	0,0025	0,01	(0.01-1)	102	5,5	19,3
111.	Foramsulfuron	0,001	0,005	(0.005-2)	82	12,8	46,0
112.	Formetanate	0,001	0,01	(0.01-2)	101	4,2	16,9
113.	Fosthiazate	0,0025	0,01	(0.01-1)	102	2,9	10,0
114.	Fuberidazole	0,001	0,005	(0.005-2)	95	2,7	15,0
115.	Halofenozide	0,002	0,01	(0.01-2)	96	2,4	20,0
116.	Hexaflumuron	0,001	0,005	(0.005-2)	102	9,0	21,0
117.	Hexytiazox	0,001	0,005	(0.005-2)	97	3,8	12,5
118.	Imazalil	0,001	0,01	(0.01-2)	98	1,7	8,4
119.	Imazapic	0,002	0,01	(0.01-2)	101	5,0	16,0
120.	Imidacloprid	0,001	0,01	(0.01-2)	99	3,2	10,0
121.	Indoxacarb	0,001	0,005	(0.005-2)	100	5,4	16,3
122.	Iodosulfuron methyl	0,0025	0,01	(0.01-1)	84	4,5	34,6
123.	Ipconazole	0,0025	0,01	(0.01-1)	98	5,4	21,0
124.	Iprovalicarb	0,001	0,005	(0.005-2)	94	3,4	16,0
125.	Isofetamid	0,002	0,01	(0.01-2)	100	2,3	17,0
126.	Isoprocarb	0,0025	0,01	(0.01-1)	103	3,0	20,0
127.	Isoprothiolane	0,0025	0,01	(0.01-1)	101	9,3	18,0
128.	Isoproturon	0,001	0,005	(0.005-2)	99	2,5	10,2
129.	Isopyrazam	0,001	0,005	(0.005-2)	98	1,7	13,9
130.	Isoxaben	0,001	0,005	(0.005-2)	110	5,0	26,0
131.	Isoxaflutole	0,001	0,005	(0.005-2)	97	14,0	29,0
132.	Isoxathion	0,001	0,005	(0.005-2)	110	6,0	26,0
133.	Lenacil	0,001	0,01	(0.01-2)	93	5,2	20,4
134.	Linuron	0,001	0,005	(0.005-2)	98	8,0	24,4
135.	Lufenuron	0,002	0,01	(0.01-2)	86	11,0	44,0
136.	Malaoxon	0,001	0,005	(0.005-2)	96	2,1	15,1

137.	Malathion	0,001	0,01	(0.01-2)	102	4,2	13,6
138.	Mandipropamid	0,001	0,005	(0.005-2)	93	2,7	16,1
139.	Metaflumizone	0,002	0,01	(0.01-2)	100	9,0	26,0
140.	Metalaxyl	0,0005	0,005	(0.005-2)	98	2,8	10,8
141.	Metamitron	0,002	0,01	(0.01-2)	94	2,7	13,7
142.	Metazachlor	0,002	0,01	(0.01-2)	94	2,3	16,0
143.	Methamidophos	0,002	0,01	(0.01-2)	93	2,6	15,7
144.	Methiocarb	0,0005	0,005	(0.005-2)	102	3,4	10,9
145.	Methiocarb sulphone	0,001	0,01	(0.01-2)	92	6,3	23,8
146.	Methiocarb sulphoxide	0,0005	0,005	(0.005-2)	93	1,8	15,4
147.	Methomyl	0,001	0,01	(0.01-2)	99	3,6	10,6
148.	Methoprotryne	0,002	0,01	(0.01-2)	112	5,0	26,0
149.	Methoxyfenozide	0,002	0,005	(0.005-2)	100	2,2	9,3
150.	Metobromuron	0,002	0,01	(0.01-2)	100	5,0	13,0
151.	Metolachlor-S	0,001	0,005	(0.005-2)	93	4,9	19,5
152.	Metosulam	0,0025	0,01	(0.01-2)	92	13,7	44
153.	Metoxuron	0,0025	0,01	(0.01-1)	101	2,4	11,4
154.	Metrafenone	0,001	0,005	(0.005-2)	99	3,2	10,1
155.	Metsulfuron-methyl	0,002	0,005	(0.005-2)	79	4,9	44,9
156.	Monocrotophos	0,001	0,005	(0.005-2)	100	3,7	13,3
157.	Monuron	0,0025	0,01	(0.01-1)	105	4,9	18,1
158.	Napropamide	0,001	0,005	(0.005-2)	98	4,1	13,8
159.	Nicosulfuron	0,001	0,005	(0.005-2)	88	11,0	32,0
160.	Nicotine	0,0025	0,01	(0.01-2)	93	8,2	26,9
161.	Nitenpyram	0,002	0,01	(0.01-2)	106	8,0	18,0
162.	Novaluron	0,001	0,005	(0.005-2)	99	14,0	35,0
163.	Omethoate	0,001	0,0025	(0.0025-2)	90	7,4	28,6
164.	Oxadixyl	0,001	0,005	(0.005-2)	97	3,2	12,5
165.	Oxamyl	0,001	0,005	(0.005-2)	93	1,8	14,6

166.	Oxycarboxin	0,0025	0,01	(0.01-1)	100	2,8	12,6
167.	Paraoxon-methyl	0,001	0,005	(0.005-2)	101	3,5	10,2
168.	Parathion	0,001	0,01	(0.01-2)	100	8,6	27,5
169.	Parathion-methyl	0,0025	0,01	(0.01-1)	100	11,7	33,7
170.	Pencycuron	0,001	0,005	(0.005-2)	96	5,7	19,7
171.	Pendimethalin	0,002	0,005	(0.005-2)	102	2,0	13,2
172.	Penflufen	0,002	0,01	(0.01-2)	117	6,0	35,0
173.	Penthiopyrad	0,0025	0,01	(0.01-1)	103	3,7	14,3
174.	Pethoxamid	0,0025	0,01	(0.01-0.5)	94	6,1	20,5
175.	Phenmedipham	0,001	0,01	(0.01-2)	97	4,4	14,1
176.	Phenthoate	0,001	0,005	(0.005-2)	96	3,0	12,0
177.	Phosmet	0,001	0,005	(0.005-2)	97	2,8	10,0
178.	Phosmet okson	0,002	0,01	(0.01-2)	109	7,7	27,6
179.	Phoxim	0,002	0,01	(0.01-2)	104	8,0	22,0
180.	Pinoxaden	0,0005	0,005	(0.005-2)	92	3,7	19,6
181.	Piperonyl butoxide	0,0025	0,01	(0.01-1)	103	2,5	16,3
182.	Prochloraz	0,001	0,005	(0.005-2)	98	2,7	8,5
183.	Prochloraz BTS 44595	0,0025	0,01	(0.01-1)	104	3,0	21,6
184.	Prochloraz BTS 44596	0,0025	0,01	(0.01-1)	101	3,4	14,8
185.	Propamocarb	0,001	0,005	(0.005-2)	80	3,8	42,1
186.	Propaquizafop	0,001	0,005	(0.005-2)	98	3,1	11,5
187.	Propoxur	0,0025	0,01	(0.01-1)	101	2,6	14,9
188.	Propoxycarbazone	0,0025	0,01	(0.01-1)	85	4,6	33,0
189.	Proquinazid	0,001	0,005	(0.005-2)	97	1,3	10,2
190.	Prosulfocarb	0,001	0,005	(0.005-2)	96	2,8	15,7
191.	Prosulfuron	0,001	0,005	(0.005-2)	94	19,0	36,0
192.	Pymetrozine	0,005	0,02	(0.02-2)	79	2,8	49
193.	Pyridaben	0,001	0,005	(0.005-2)	93	2,7	15,8
194.	Pyridafol	0,002	0,01	(0.01-2)	79	3,9	47

195.	Pyrifenox	0,002	0,01	(0.01-2)	92	8,0	27,0
196.	Pyriproxyfen	0,0025	0,01	(0.01-1)	104	2,7	16,7
197.	Pyroquilon	0,0025	0,01	(0.01-1)	101	2,3	11,5
198.	Pyroxsulam	0,001	0,005	(0.005-2)	94	7,0	18,0
199.	Quinclorac	0,002	0,01	(0.01-2)	114	17,0	38,0
200.	Quinoclanmine	0,0025	0,01	(0.01-1)	105	5,2	26,8
201.	Quizalofop-ethyl	0,001	0,005	(0.005-2)	96	2,2	11,8
202.	Rimsulfuron	0,001	0,01	(0.01-2)	84	4,2	44,7
203.	Rotenone	0,0025	0,01	(0.01-1)	103	3,5	27,0
204.	Saflufenacil	0,001	0,005	(0.005-2)	102	12,0	26,0
205.	Silthiofam	0,001	0,005	(0.005-2)	95	6,2	20,4
206.	Spinetoram	0,0025	0,01	(0.01-1)	102	2,6	22,0
207.	Spinosyn A	0,001	0,005	(0.005-2)	94	2,5	13,5
208.	Spinosyn D	0,001	0,005	(0.005-2)	95	1,9	13,7
209.	Spirodiclofen	0,001	0,005	(0.005-2)	92	2,5	17,7
210.	Spirotetramat	0,001	0,005	(0.005-2)	95	3,5	15,8
211.	Spirotetramat enol	0,001	0,005	(0.005-2)	78	3,9	48,1
212.	Spirotetramat enol-glucoside	0,002	0,005	(0.005-2)	85	3,9	42,0
213.	Spirotetramat ketohydroxy	0,001	0,005	(0.005-2)	92	9,4	18,7
214.	Spirotetramat monohydroxy	0,001	0,005	(0.005-2)	94	5,4	19,4
215.	Spiroxamine	0,001	0,005	(0.005-2)	94	2,7	14,4
216.	Sulfometuron methyl	0,002	0,005	(0.005-2)	77	3,5	47,4
217.	Sulfosulfuron	0,0025	0,01	(0.01-1)	90	4,4	25,8
218.	Sulfoxaflor	0,002	0,01	(0.01-2)	97	12,0	10,0
219.	Tebuconazole	0,002	0,01	(0.01-2)	97	7,3	24,4
220.	Tebufenozide	0,001	0,005	(0.005-2)	93	2,5	17,1
221.	Tebufenpyrad	0,001	0,005	(0.005-2)	99	5,4	17,0
222.	Teflubenzuron	0,005	0,01	(0.01-2)	93	6,7	23,7
223.	Tepraloxydim	0,005	0,01	(0.01-2)	97	6,5	19,6

224.	Terbufos	0,0025	0,01	(0.01-0.5)	94	6,1	21,0
225.	Terbufos oxon	0,002	0,01	(0.01-2)	85	3,0	32,0
226.	Terbufos sulphone	0,0025	0,01	(0.01-0.5)	93	5,9	21,9
227.	Terbufos sulphoxide	0,001	0,005	(0.005-2)	93	7,8	26,7
228.	Terbuthylazine	0,001	0,005	(0.005-2)	97	2,2	9,4
229.	Thiabendazole	0,001	0,005	(0.005-2)	93	1,7	15,5
230.	Thiacloprid	0,001	0,005	(0.005-2)	98	2,0	9,7
231.	Thiamethoxam	0,001	0,005	(0.005-2)	97	2,2	12,5
232.	Thiencarbazone-methyl	0,001	0,005	(0.005-2)	97	14,0	33,0
233.	Thifensulfuron-methyl	0,002	0,01	(0.01-2)	76	4,0	49,0
234.	Thiodicarb	0,0005	0,005	(0.005-2)	93	2,7	15,7
235.	Thiometon	0,0025	0,01	(0.01-1)	96	7,2	22,0
236.	Thiophanate-methyl	0,001	0,005	(0.005-2)	97	1,9	10,6
237.	Tolfenpyrad	0,002	0,01	(0.01-2)	80	2,0	39,0
238.	Topramezone	0,002	0,01	(0.01-2)	98	5,0	11,0
239.	Tralkoxydim	0,0025	0,01	(0.01-1)	92	3,3	22,0
240.	Trichlorfon	0,002	0,01	(0.01-2)	96	9,0	24,0
241.	Tricyclazole	0,0025	0,01	(0.01-1)	97	2,4	15,8
242.	Tridemorph	0,002	0,01	(0.01-2)	89	7,0	31,0
243.	Triflumizole	0,002	0,01	(0.01-2)	88	3,6	26,0
244.	Triflumuron	0,002	0,01	(0.01-2)	100	13,0	25,0
245.	Triflusulfuron methyl	0,0025	0,01	(0.01-0.5)	83	4,0	35,5
246.	Triticonazole	0,001	0,01	(0.01-2)	105	8,6	28,9
247.	Tritosulfuron	0,002	0,01	(0.01-2)	89	4,0	25,0
248.	Zoxamide	0,001	0,005	(0.005-2)	95	3,2	15,6
**SUB-METHOD 1**							
1.	Cyhexatin	0,001	0,005	0.005-1	79,2	10,03	28,37
2.	Dithianon	0,001	0,005	0.005-0.5	102,3	3,96	11,21
3.	Dodine	0,001	0,005	0.005-1	85,6	7,77	21,97

4.	Fentin	0,001	0,0025	0.0025-1	86,1	5,76	16,29
5.	Flonicamid	0,001	0,005	0.005-1	86,5	2,64	7,48
6.	TFNA	0,001	0,005	0.005-1	84,7	5,34	15,12
7.	TFNA-AM	0,001	0,005	0.005-1	83,9	10,25	29,00
8.	TFNG	0,001	0,005	0.005-1	84,07	3,58	10,12
9.	Fenbutatin oxide	0,001	0,005	0.005-1	107,8	4,12	11,65
**SUB-METHOD 2**							
1.	2,4,5-T	0,0025	0,01	(0.01 - 0.5)	98	3,93	11
2.	2,4,5-TP (Fenoprop)	0,0025	0,01	(0.01 - 0.5)	96	3,35	9
3.	2,4-D	0,0025	0,01	(0.01 - 0.5)	102	2,90	8
4.	2,4-DB	0,0025	0,01	(0.01 - 0.5)	98	5,42	15
5.	Acibenzolar acid	0,0025	0,01	(0.01 - 0.5)	89	3,19	9
6.	Acifluorfen	0,0025	0,01	(0.01 - 0.5)	88	5,30	15
7.	Aminopyralid	0,0025	0,01	(0.01 - 0.5)	81	4,16	12
8.	Bentazone	0,0025	0,01	(0.01 - 0.5)	95	3,88	11
9.	Bromacil	0,0025	0,01	(0.01 - 0.5)	102	6,70	19
10.	Bromoxynil	0,0025	0,01	(0.01 - 0.5)	94	4,75	13
11.	Clopyralid	0,0025	0,01	(0.01 - 0.5)	85	2,91	8
12.	Dichlorprop	0,0025	0,01	(0.01 - 0.5)	98	3,33	9
13.	Diflufenzopyr	0,0025	0,01	(0.01 - 0.5)	91	5,20	15
14.	Dicamba	0,0025	0,01	(0.01 - 0.5)	93	3,68	10
15.	Diclofop	0,0025	0,01	(0.01 - 0.5)	92	6,78	19
16.	Fenoxaprop-P	0,0025	0,01	(0.01 - 0.5)	93	5,52	16
17.	Fluazifop	0,0025	0,01	(0.01 - 0.5)	98	3,16	9
18.	Fluroxypyr	0,0025	0,01	(0.01 - 0.5)	92	6,17	17
19.	Haloxyfop	0,0025	0,01	(0.01 - 0.5)	95	3,33	9
20.	Imazamox	0,0025	0,01	(0.01 - 0.5)	98	2,20	6
21.	Imazapyr	0,0025	0,01	(0.01 - 0.5)	81	3,30	13
22.	Imazethapyr	0,0025	0,01	(0.01 - 0.5)	92	4,42	13

23.	Ioxynil	0,0025	0,01	(0.01 - 0.5)	96	3,82	11
24.	MCPA	0,0025	0,01	(0.01 - 0.5)	94	4,15	12
25.	MCPB	0,0025	0,01	(0.01 - 0.5)	99	3,43	10
26.	Mecoprop	0,0025	0,01	(0.01 - 0.5)	95	4,19	12
27.	Picloram	0,0025	0,01	(0.01 - 0.5)	88	3,82	11
28.	Triasulfuron	0,0025	0,01	(0.01 - 0.5)	99	2,66	8
29.	Tribenuron Methyl	0,0025	0,01	(0.01 - 0.5)	83	7,63	22
30.	Triclopyr	0,0025	0,01	(0.01 - 0.5)	97	4,98	14
31.	Trinexapac	0,0025	0,01	(0.01 - 0.5)	86	5,19	15
32.	Quinmerac	0,0025	0,01	(0.01 - 0.5)	81	4,70	13
33.	Quizalofop	0,0025	0,01	(0.01 - 0.5)	98	4,17	12
**DITIOCARBAMATES**							
1.	Ditiocarbamates	0,002	0,005	0,005-75	88	10	31,1
**QuPPE**							
1.	Glyphosate	0,0025	0,01	(0.01 - 10)	106	4,3	12,3
2.	Trimethyl-sulfonium cation, resulting from the use of glyphosate	0,0025	0,01	(0.01 - 10)	109	3,2	9,0
3.	Ethephon	0,0025	0,01	(0.01 - 10)	104	2,2	6,2
4.	Glufosinate	0,005	0,025	(0.025 - 10)	96,1	4,17	11,8
5.	3-[hydroxy(methyl)phosphinoyl]propionic acid (MPP)	0,005	0,025	(0.025 - 10)	99	1,81	5,1
6.	N-acetyl-glufosinate (NAG)	0,005	0,025	(0.025 - 10)	92,7	3	8,5
7.	Phosphonic acid and its salts expressed as phosphonic acid	0,02	0,1	(0.1 - 20)	87	2,82	8,0
8.	Fosetyl	0,002	0,01	(0.01 - 20)	98	3,26	9,0
9.	Bromide ion	0,05	0,2	(0.2 - 20)	94	3,74	11,0
10.	Chlorate	0,002	0,01	(0.01 - 20)	93	3,25	9,0
11.	Perchlorate	0,002	0,01	(0.01 - 20)	100	5,75	16,0
